# Hybrid adaptive PID control strategy for UAVs using combined neural networks and fuzzy logic

**DOI:** 10.1371/journal.pone.0331036

**Published:** 2025-08-29

**Authors:** Nigatu Wanore Madebo

**Affiliations:** Information Network Security Administration (INSA), Aerospace Division, Addis Ababa, Ethiopia; Embry-Riddle Aeronautical University, UNITED STATES OF AMERICA

## Abstract

This paper presents a novel hybrid combined neural network and fuzzy logic adaptive proportional, integral, and derivative(NNPID+FPID) control strategy that integrates neural networks and fuzzy logic for optimizing Unmanned Aerial Vehicle(UAV) dynamics by tuning the gains of a PID controller. The proposed approach leverages the strengths of each technique by applying neural networks to fine-tune the *y* and *ψ* states, while fuzzy logic enhances the performance of *x*, *z*, *ϕ*, and *θ* dynamics. A single-layer neural network with 10 hidden neurons is utilized to adjust PID gains for the *y* and *ψ* states using proportional, integral, and derivative errors ($e_{p},e_{i},e_{d}$) as inputs. The weights are updated through a gradient descent algorithm minimizing the mean squared error, with a nonlinear sigmoid activation function ensuring adaptability. Concurrently, fuzzy logic employs heuristic rules to dynamically tune PID gains for the remaining states, based on input errors and their derivatives. Membership functions map inputs to gains to ensure real-time adaptability. The hybrid method outperforms standalone neural network(NNPID) and fuzzy logic(FPID) approaches by significantly improving trajectory tracking performance and overall UAV control efficiency. This work demonstrates the effectiveness of combining neural networks and fuzzy logic to address the multi-dimensional control challenges of UAV systems.

## 1 Introduction

Over the past decade, the use of multirotor drones for industrial applications has increased significantly, including tasks such as disaster inspection, bridge assessment, precision agriculture, remote farming, search and rescue missions, and transportation [1]. Quadrotors offer substantial commercial potential; however, the number of sensors and devices mounted on each aircraft varies depending on the specific application. Consequently, manufacturers must individually configure control parameters for each aircraft, even for the same base model. This process requires a significant number of skilled pilots and is often conducted outdoors, where adverse weather can substantially extend processing time. A simulation environment enabling parameter adjustment and direct deployment to real quadrotors would greatly enhance the efficiency of the manufacturing process.In recent years, the UAV industry’s growth has driven increased demand and broader applications for UAVs. However, the high operational complexity and cost of UAV pilots have elevated the need for greater autonomy. Motion planning in complex and unknown environments has emerged as a critical technology for achieving UAV autonomy [2,3]. Quadrotors face challenges in control design due to their unstable dynamics and sensitivity to external disturbances, necessitating robust and efficient control strategies to ensure stability and optimal performance [4]. While classical Proportional-Integral-Derivative (PID) controllers are widely used for their simplicity and effectiveness, their performance often declines in nonlinear or highly dynamic environments typical of quadrotors. To address these limitations, Fuzzy Logic Controllers (FLCs) have been introduced. FLCs excel in handling uncertainties and nonlinearities, making them ideal for complex systems like quadrotors [5]. Combining fuzzy logic with traditional PID controllers creates Fuzzy PID controllers, which utilize the advantages of heuristic decision-making and classical control to enhance overall performance [6–8]. The neural network-based self-tuning PID controller demonstrates strong anti-interference capabilities and fast convergence rates [9–11]. Hybrid Fuzzy Neural Network controllers provide improved adaptability and robustness in dynamic environments [12–18].

### 1.1 Related work

The control of quadrotor UAVs has advanced significantly as researchers have addressed challenges associated with their nonlinear and inherently unstable dynamics. Traditional control methods, such as proportional-integral-derivative (PID) controllers, are widely adopted due to their simplicity. However, they often lack the robustness and adaptability needed to handle complex, real-world environments characterized by varying system parameters and unpredictable external disturbances. A control algorithm combining fuzzy logic with PID and self-optimal regulation is presented in [19], offering enhanced adaptability. Similarly, the experimental application of a fuzzy PID controller for multi-joint robotic arm control is investigated in [20]. The study demonstrates the controller’s effectiveness in managing complex dynamics while maintaining system stability. The work in [21] introduces a hybrid fractional-order fuzzy PID (FOPID) controller tailored for fractional-order systems. The controller replaces the proportional term of the FOPID structure with an interval type-2 (IT2) fuzzy neural network, while retaining the fractional-order integral and derivative components. To improve performance, the fractional-order derivative is applied to the process output, reducing abrupt changes in the set-point. Additionally, the IT2 fuzzy neural network parameters are adaptively tuned in real-time using the gradient descent algorithm, enabling enhanced adaptability to system uncertainties and improved time response. Reference [22] describes a neural network-based feedback control method designed to enhance the control precision and tracking speed of permanent magnet brushless motors under command control. This approach adaptively adjusts the PID controller parameters in real-time based on electromechanical output signals, ensuring feedback control tailored to the motor’s operational conditions. Experimental results show that this method significantly improves real-time performance, dynamic load response, and tracking accuracy, achieving low-distortion current waveforms. Furthermore, this approach has been successfully implemented in the development of commercial products.

The study in [23] proposes a fuzzy neural network control methodology with feed-forward compensation, aimed at achieving precise control of a distributed electric tail rotor system. A comprehensive analysis of the helicopter’s motion characteristics using six-degree-of-freedom physical modeling serves as the foundation for the control strategy. By combining fuzzy control and neural networks, the methodology balances stability and dynamic response in the tail rotor system.The feed-forward compensation mechanism preemptively predicts posture changes in the tail rotor system, enabling rapid responses to external disturbances. Additionally, the control strategy dynamically adjusts the proportional-integral (PI) parameters using a fuzzy neural network algorithm. Simulation results validate the effectiveness of the approach, demonstrating improved yaw angle control accuracy under varying flight conditions compared to conventional PID and fuzzy PID control methods. In reference [24], the authors proposes new fuzzy controller design methods, which retain strengths of traditional controller and controller with modifiable factors. The proposed fuzzy controller also adopts a four-layer neural network to optimize the control rules of compromise to improve control precision and system robustness. Finally, the author proves the proposed fuzzy controller has the advantages of higher control precision and smaller transition. In reference [25], proposes a novel fuzzy proportional-integral-derivative (PID)-type iterative learning control (ILC) method to enhance the performance of quadrotor UAVs. The approach integrates the robustness of ILC, known for its ability to manage disturbances and uncertainties, with fuzzy control to improve adaptability. A new control law, based on the PID-ILC algorithm, addresses the chattering issue often encountered when external disturbances affect ILC. Fuzzy control dynamically adjusts the PID parameters of the learning gain matrices, mitigating the impact of uncertainties and enhancing control precision. Stability analysis using Lyapunov’s theory confirms the stability of the proposed control scheme. Simulations conducted in the Gazebo environment demonstrate significant improvements in the trajectory tracking performance of quadrotor UAVs. In reference [26], proposes a fuzzy proportional-integral-derivative (FPID) controller for stabilizing and tracking the desired trajectory of a quadcopter UAV. The controller is applied to both the inner and outer loops to regulate the UAV’s attitude and position, respectively. By adaptively adjusting system inputs based on tracking errors, the FPID controller demonstrates superior performance compared to conventional PID controllers. Simulation results using MATLAB/Simulink show that the FPID controller reduces position errors by 87% and attitude errors by 70% relative to the traditional PID approach.In reference [27],authors propose a learning model-free control method for accurate trajectory tracking and safe landing of unmanned aerial vehicles (UAVs). A realistic scenario is considered where the UAV commutes between stations at high-speeds, experiences a single motor failure while surveying an area, and thus requires to land safely at a designated secure location. The challenge is viewed solely as a control problem. A hybrid control architecture – an artificial neural network (ANN)-assisted proportional-derivative controller – is able to learn the system dynamics online and compensate for the error generated during different phases of the considered scenario: fast and agile flight, motor failure, and safe landing. Firstly, it deals with unmodelled dynamics and operational uncertainties and demonstrates superior performance compared to a conventional proportional-integral-derivative controller during fast and agile flight. Secondly, it behaves as a fault-tolerant controller for a single motor failure case in a coaxial hexacopter thanks to this authors for proposing sliding mode control theory-based learning architecture. Lastly, it yields reliable performance for a safe landing at a secure location in case of an emergency condition. The tuning of weights is not required as the structure of the ANN controller starts to learn online, each time it is initialised, even when the scenario changes – thus, making it completely model-free. Moreover, the simplicity of the neural network-based controller allows for the implementation on a low-cost low-power onboard computer.Authors demonstrated that the real-time experiments show that the proposed controller outperforms the conventional controller. In reference [28] author proposes, a deep learning neural network—fuzzy-tuned proportional integral derivative (PID) speed controller for brushless DC motor. Deep learning architecture is designed for the multi-layer perceptron network, and the output from the neural module fires the rules of the fuzzy inference system mechanism. The parameters of deep perceptron neural network (DPNN) are tuned for near optimal solutions using the unified multi-swarm particle swarm optimization, and in turn the optimized DPNN selects the parameters of the fuzzy inference system. Deep learning neural network with the fuzzy inference system tunes the gain values of the PID controller and performs an effective speed regulation. The performance characteristics of the designed speed controller are tested for a step change in input speed and also for impulsive load disturbances. Further, the stability analysis of the controller is investigated with the Lyapunov stability criterion by deriving the positive definite functions. The weight parameters of DPNN model and the number of rules of fuzzy system are tuned for their near optimal solutions using multi-swarm particle swarm optimization. From the results, it is well proven that the proposed controller is more stable and guarantees consistent performance than other considered controllers in all aspects, the design methodologies outperform other controller designs from the literature. In reference [29] author suggests the use of neural network observers based on fuzzy auxiliary sliding mode control for the fault estimation and isolation of quadrotor unmanned aerial vehicle sensors.The fuzzy auxiliary sliding-mode-control-based adaptive approach for neural network observer is used for the approximation, reconstruction, and isolation of unknown faults by utilizing the multi-layer neural network. The neural network weight parameters are updated adaptively by using the fuzzy auxiliary sliding-mode-control approach. In conventional neural networks, the gradient descent approach-based back-propagation procedures are adapted for the training of the neural network. The nonlinear controller such as the fuzzy auxiliary sliding-mode-control method is adapted for neural network online training, in which the fuzzy auxiliary sliding-mode-control is used as the learning approach; the neural network is used as a control process that calculates the stable and dynamic learning rates. In reference [30] authors introduce a robust hybrid control system, including a linear Strictly Negative Imaginary (SNI) controller and an adaptive nonlinear Neural-Fuzzy control law, to enable high-precision trajectory tracking tasks for a quadcopter drone. Based on a parallel form, both controllers are able to enhance the transient performance, the system response, and the robustness of the quadcopter controllers. Also author proposed a linear time-invariant SNI UAV dynamic model, in combination with an online adaptive residual nonlinear model using the neural network identification, to model the natural behavior of a quadcopter system. Through a series of numerical simulations, this literature highlights the effectiveness of hybrid controllers in the face of some parameter variations, such as nonlinear aerodynamic models and exogenous disturbances (e.g., wind gusts). This body of work highlights diverse approaches that integrate neural networks, fuzzy logic, and advanced control techniques to address the challenges of nonlinear and dynamic systems, paving the way for robust and adaptable control methodologies. Efficient reference tracking for a quadrotor UAV using a neural network PID(NNPID) and fuzzy PID(FPID) standalone controller depends on accurately tuning the PID parameter gains to optimize controller performance in all dynamic states. However, NNPID and FPID controllers often fall short in managing the nonlinear dynamics of quadrotor systems in some states. To overcome this limitation, this study proposes a trajectory-tracking control method based on a hybrid fuzzy neural network-tuned PID (NNPID+FPID) controller for nonlinear quadrotors. This approach integrates real-time adjustment of PID parameter gains using fuzzy logic and neural networks, enabling precise and adaptive trajectory control.

The literature reveals a strong focus on combining fuzzy logic, neural networks, and intelligent control to improve UAV robustness. However, standalone NNPID and FPID systems struggle with full-state nonlinear tracking. To address this, our proposed hybrid NNPID+FPID approach offers adaptive PID gain tuning across all UAV dynamic states for enhanced tracking performance (Table 1).

**Table 1:** Summary of related work and identified research gaps.

Ref.	Approach	Key Contributions	Research Gap
[19]	Fuzzy PID with self-optimal regulation	Enhances adaptability through fuzzy rule optimization.	Not validated on nonlinear UAV dynamics.
[20]	Fuzzy PID for robotic arms	Demonstrates effective control in multi-joint mechanisms.	Not tested in aerial or real-time environments.
[21]	FOPID with IT2 Fuzzy NN	Improves time response using fractional-order terms.	Complexity limits real-time UAV implementation.
[22]	NN-tuned PID for motors	Enables real-time PID gain tuning via feedback.	Focused on motor level; not entire UAV dynamics.
[23]	Fuzzy NN with feedforward	Enhances tail rotor stability in rotorcraft systems.	Not generalized to quadrotor UAVs.
[24]	NN-optimized fuzzy controller	Increases robustness via NN-tuned fuzzy rules.	Lacks lightweight real-time implementation.
[25]	Fuzzy PID with ILC	Improves control under disturbances.	Prone to chattering; lacks UAV-specific tuning.
[26]	Dual-loop FPID control	Reduces UAV position and attitude errors.	Uses fixed fuzzy rules; lacks full adaptability.
[27]	ANN-PD for UAV emergencies	Adapts in-flight to motor failure and landing.	Stability under fast-changing failures unproven.
[28]	Deep NN + fuzzy PID (PSO)	Provides optimized gains for speed control.	High complexity; not ideal for UAV onboard use.
[29]	Fuzzy SM NN observers	Detects sensor faults using adaptive control.	Designed for fault estimation, not full control.
[30]	SNI + Neural-Fuzzy hybrid	Combines linear/nonlinear models for UAVs.	Not easily generalizable across UAV types.

### 1.2 Contributions

The main contributions of this paper are summarized below.

1. **Development of a Hybrid Control Strategy:** A novel hybrid control framework (NNPID+FPID) is introduced, combining neural networks and fuzzy logic to optimize the Proportional-Integral-Derivative (PID) controller gains for UAV dynamics.
2. **State-Specific Tuning:** Neural networks are utilized to adaptively tune PID gains for the *y* and *ψ* states, addressing the need for precise trajectory tracking in these dynamics. Fuzzy logic is employed for real-time tuning of PID gains for *x*,*z*,*ϕ*,*θ* states, ensuring adaptability and stability for these dynamics.
3. **Integration of Neural Network and Fuzzy logic Approaches:** By leveraging the precision of neural networks and the heuristic adaptability of fuzzy logic, the proposed hybrid strategy overcomes the individual limitations of standalone neural network-based PID (NNPID) and fuzzy logic-based PID (FPID) approaches.
4. **Enhanced Control Performance:** The hybrid approach demonstrates superior trajectory tracking and system stability across all six UAV dynamic states compared to traditional NNPID and FPID methods.
5. **Efficient Neural Network Design:** A single-layer neural network with 10 hidden neurons and a nonlinear sigmoid activation function is designed for computational efficiency and adaptability.
6. **Real-Time Tuning with Fuzzy Logic:** The fuzzy logic controller uses heuristic rules and membership functions for online tuning of PID gains, ensuring adaptability to real-time UAV dynamics.
7. **Experimental Validation:** The proposed hybrid control strategy is validated through simulations and performance metrics, showing significant improvements in UAV tracking accuracy and control efficiency compared to standalone methods.
8. **Comparative Analysis:** Based on tracking performance the strength and limitation of NNPID, FPID, and hybrid approach(NNPID+FPID) controllers for UAV were studied.

### 1.3 Methodology

The proposed methodology aims to develop a hybrid adaptive PID control system based on neural networks and fuzzy logic for Unmanned Aerial Vehicle (UAV) dynamics. The approach consists of the following steps:

**1) Problem Analysis and System Modeling:** The six degrees of freedom of the UAV (*x*, *y*, *z*, *ϕ*, *θ*, *ψ*) are modeled using dynamic equations. The control objectives include stable trajectory tracking, minimization of tracking errors, and robustness against external disturbances and uncertainties. A mathematical model of the UAV is formulated to serve as the plant for the control system.

**2) Neural Network PID (NNPID) Controller Design:** A single-layer feedforward neural network with 10 hidden neurons is developed. The inputs are proportional (*e*_*p*_), integral (*e*_*i*_), and derivative (*e*_*d*_) errors, while the outputs are the adaptive PID gains (*K*_*p*_, *K*_*i*_, *K*_*d*_) for the *y* and *ψ* states. The network is trained online using a gradient descent algorithm with a sigmoid activation function. Weights and biases are updated in real time until convergence is achieved. The trained network is integrated into the control loop for adaptive tuning.

**3) Fuzzy Logic PID (FPID) Controller Design:** A fuzzy inference system is designed using heuristic rules to map input errors *e*(*t*) and their derivatives $\frac{de(t)}{dt}$ to PID gains. This controller is applied for online tuning of the *x*, *z*, *ϕ*, and *θ* states, ensuring adaptability and robustness.

**4) Hybrid Controller Integration:** The NNPID and FPID controllers are combined into a hybrid architecture. The NNPID component handles precise trajectory tracking of the *y* and *ψ* states, while the FPID component manages the remaining states (*x*, *z*, *ϕ*, *θ*), enabling real-time adaptation and improved robustness.

**5) Performance Evaluation:** The proposed system is tested under various scenarios, including trajectory tracking along predefined paths, disturbance rejection (e.g., wind gusts), and parameter variations (e.g., changes in weight or motor efficiency). Performance is evaluated using standard metrics such as tracking error and system stability.

**6) Comparative Analysis and Expected Outcomes:** Simulation results are used to compare the individual and hybrid controllers. While standalone NNPID and FPID controllers exhibit limitations in handling nonlinear UAV dynamics, the hybrid approach demonstrates superior performance. Benefits include faster response, reduced steady-state error and overshoot, and increased robustness to disturbances and model variations.

**7) Recommendations:** The findings support the adoption of hybrid intelligent control strategies for real-world UAV applications, offering a balanced trade-off between precision and adaptability in complex flight environments (Table 2).

**Table 2:** Summary of methodology.

Step	Phase	Description
1	Problem Analysis & System Modeling	Analyze UAV dynamics (*x*, *y*, *z*, *ϕ*, *θ*, *ψ*). Define control objectives: tracking and robustness. Develop a mathematical model of the UAV.
2	NNPID Controller Design	Implement a single-layer feedforward neural network (10 hidden neurons). Inputs: *e*_*p*_, *e*_*i*_, *e*_*d*_. Outputs: *K*_*p*_, *K*_*i*_, *K*_*d*_ for *x*, *y*, *z*, *ϕ*, *θ*, *ψ* states.
3	Neural Network Training	Use gradient descent to minimize loss. Apply sigmoid activation function. Perform online weight and bias updates for continuous learning.
4	NNPID Implementation	Integrate the trained neural network into the control loop for real-time adaptive tuning of all six state variables.
5	FPID Controller Design	Formulate fuzzy logic rules based on *e*(*t*) and $\frac{de(t)}{dt}$. Map these to *K*_*p*_, *K*_*i*_, *K*_*d*_ gains for *x*, *y*, *z*, *ϕ*, *θ*, *ψ* states.
6	Analysis of Limitations (Standalone Controllers)	FPID offers good tracking for *x*, *z*, *ϕ*, *θ*, while NNPID provides accurate control for *y*, *ψ* states. Limitations exist in standalone performance.
7	Hybrid Controller Integration	Combine NNPID (for *y*, *ψ*) and FPID (for *x*, *z*, *ϕ*, *θ*) into a unified hybrid system. Enhances tracking precision and robustness.
8	Performance Evaluation	Test hybrid controller under trajectory tracking, external disturbances, and system parameter variations. Assess using error, stability, and robustness metrics.
9	Comparative Analysis	Compare individual (NNPID, FPID) and hybrid controller performance. Highlight respective strengths and weaknesses.
10	Expected Outcomes	Demonstrate hybrid controller’s superiority: faster response, reduced error, improved resilience to disturbances.
11	Recommendations	Propose real-world UAV deployment of hybrid intelligent control for enhanced performance and adaptability.

The remainder of this article is organized as follows: Sect 2 outlines the modeling of the quadrotor UAV, Sect 3 presents the problem statement and the proposed solution, Sect 4 discusses the design of the controllers, Sect 5 provides the simulation results, while Sect 6 addresses the disturbance rejection and robustness against parameter variation capabilities for standalone NNPID and FPID controllers, Sect 7 simulation of the system using hybrid(NNPID+FPID), NNPID, and FPID controllers to assess standalone and hyprid aproach responses for quadrotor trajectory control, Sect 8 addresses the disturbance rejection and robustness against parameter variation capabilities for standalone NNPID, FPID and hyprid(NNPID+FPID)controllers, Sect 9 compares the controllers based on performance criteria, Finally, Sect 10 concludes the paper by summarizing the key findings and suggesting directions for future research.

## 2 System modeling

**Fig 1:**
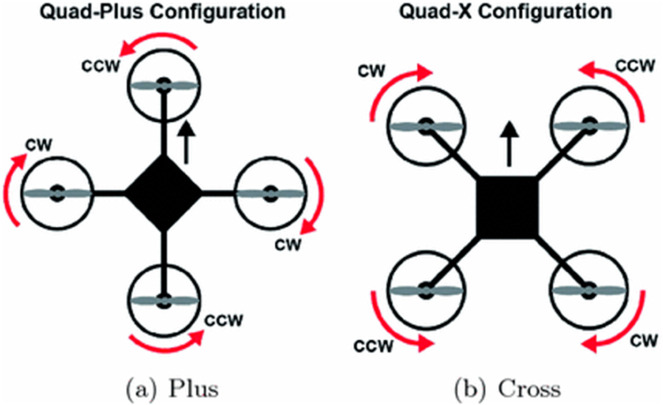
Quadrotor configurations and motion relative to propeller speed [32].

The modeling of a quadrotor system is a critical step in understanding its behavior and developing effective control strategies. Quadrotors, classified as unmanned aerial vehicles (UAVs) with four rotors, exhibit complex dynamics arising from the interaction of aerodynamics, mechanics, and control systems. This section outlines the fundamental concepts necessary for the system modeling of a quadrotor. This paper investigates the trajectory tracking control challenges associated with the ‘X’ (cross) configuration quadrotor UAV. The propulsion system of this UAV consists of four motors, as illustrated in Fig 1, where adjustments in rotor speeds are utilized to control both attitude and position [32].

**Proposed Model:** This study adopts the ‘X’ (cross) configuration for the quadrotor model. The primary advantage of this configuration lies in its superior stability compared to the plus configuration, as the cross configuration utilizes two rotors to stabilize and guide the quadrotor’s movements [35].

**Fig 2:**
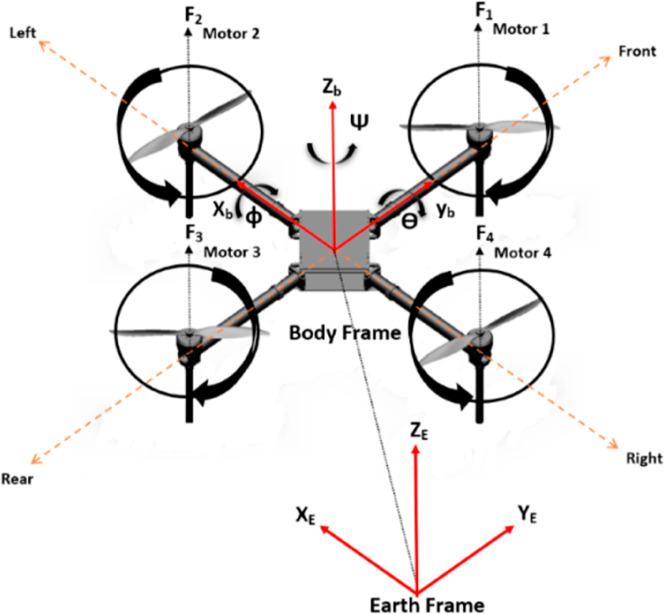
Reference frame [36].

The subsequent section focuses on Dynamics and Kinematics,of the quadrotor system. This analysis considers variables such as rotor thrust, torque, and the overall motion of the system in three-dimensional space. In the realm of the Rigid Body Control Model, we introduce how the quadrotor responds to external inputs and disturbances. This involves a comprehensive understanding of the forces and moments acting on the vehicle system. The subsequent segment encompasses the presentation of mathematical equations dictating the quadrotor’s motion. This includes translational and rotational dynamics, along with the presentation of equations of motion and their derivations from a reference frame in Fig 2. To precisely depict the position and attitude of the quadrotor UAV, the Earth-fixed coordinate system is utilized as (${\text{o}}^{\text{E}}$, ${\text{x}}^{\text{E}}$, ${\text{y}}^{\text{E}}$, ${\text{z}}^{\text{E}}$) and the body coordinate system(${\text{o}}^{\text{B}}$, ${\text{x}}^{\text{B}}$, ${\text{y}}^{\text{B}}$, ${\text{z}}^{\text{B}}$) are introduced.

The definition of the body structure and the coordinate system is shown in Fig 2. Modeling the quadrotor UAV is complex, to simplify the model establishment, the following assumptions are considered [33,34,36].

1. **Rigid and symmetrical structure:** This assumption implies that the quadrotor has a fixed shape and its mass is uniformly distributed.
2. **Center of gravity:** Assuming that the quadrotor’s center of gravity coincides with the body-fixed frame origin simplifies the analysis of the system dynamics. This assumption eliminates the need to consider the translation of the center of gravity.
3. **Rigid propellers:** Assuming that the propellers are rigid simplifies the modeling of the forces and moments generated by the propellers.
4. **Thrust and drag force:** The assumption that the thrust and drag forces are proportional to the square of the propeller’s speed.
5. **Motor dynamics:** Neglecting the motor’s resistance and inductance implies that the motor dynamics are significantly faster than the dynamics of the quadrotor itself. This assumption simplifies the modeling process by assuming that the motor instantaneously responds to changes in voltage and produces the corresponding output speed.

The orientation of the quadrotor is given by the rotation matrix *R* which depends on the three Euler angles (*ϕ*,*θ*,*ψ*).

$$R(x,\phi )=\left[\begin{array}{ccc} 1 & 0 & 0\\ 0 & cos\phi & sin\phi \\ 0 & -sin\phi & cos\phi \end{array}\right]$$ (1)

$$R(y,\theta )=\left[\begin{array}{ccc} cos\theta & 0 & -sin\theta \\ 0 & 1 & 0\\ sin\theta & 0 & cos\theta \end{array}\right]$$ (2)

$$R(z,\psi )=\left[\begin{array}{ccc} cos\psi & sin\psi & 0\\ -sin\psi & cos\psi & 0\\ 0 & 0 & 1 \end{array}\right]$$ (3)

$$\left[\begin{array}{c} \frac{dx}{dt}\\ \frac{dy}{dt}\\ \frac{dz}{dt} \end{array}\right]={\;\;}_{B}^{E}R\left[\begin{array}{c} u\\ v\\ w \end{array}\right]$$ (4)

**Transfer matrix (*T*)**

$A^{E}=[\phi ,\theta ,\psi {]}^{T}$ is in Inertial frame and Angular Velocity $\left(\omega^{B}=[p,q,r{]}^{T}\right)$ is in Body frame. To relate the two, Transfer matrix(T) is used as [34,36]:

$${\dot{A}}^{E}=T\omega^{B}$$ (5)

$$\left[\begin{array}{c} \dot{\phi }\\ \dot{\theta }\\ \dot{\psi } \end{array}\right]=T\left[\begin{array}{c} p\\ q\\ r \end{array}\right]$$ (6)

$$T=\left[\begin{array}{ccc} 1 & \sin(\phi )(\tan(\theta ) & \cos(\phi )\tan(\theta )\\ 0 & \cos(\phi ) & -\sin(\phi )\\ 0 & \sin(\phi )\sec(\theta ) & \cos(\phi )\sec(\theta ) \end{array}\right]$$ (7)

$$T^{-1}=\left[\begin{array}{ccc} 1 & 0 & -\sin(\theta )\\ 0 & \cos(\phi ) & \sin(\phi )\cos(\theta )\\ 0 & -\sin(\phi ) & \cos(\phi )\cos(\theta ) \end{array}\right]$$ (8)

**Newton-Euler method formalism.** The flight dynamics of the quadrotor UAV will be modeled using the Newton-Euler method [35–37]. The equations of motion, which combine the translational and rotational parameters which is described in Table 3 of the 6DOF system (consisting of 3 position and 3 angular orientation variables), are derived based on Euler’s two laws of motion – Newton’s second law and Newton’s first law (also known as the law of inertia) [38]. The formulation commonly involves the following parameters [35].

- Linear Velocity $\left(V^{B}=[u,v,w{]}^{T}\right)$
- Angular Velocity $\left(\omega^{B}=[p,q,r{]}^{T}\right)$
- Orientation Vector $\left(A^{E}=[\phi ,\theta ,\psi {]}^{T}\right)$
- Position Vector $\left(L^{E}=[x,y,z{]}^{T}\right)$

$$\left[\begin{array}{c} \text{ Roll rate }\\ \text{ Pitch rate }\\ \text{ Yaw rate } \end{array}\right]={\left[\begin{array}{c} p\\ q\\ r \end{array}\right]}_{B}$$ (9)

**Table 3:** Parameters description [34].

Parameters	Description	Units
$\left[\begin{array}{ccc} x & y & z \end{array}\right]$	Linear position vector	m
$\left[\begin{array}{ccc} \phi & \theta & \psi \end{array}\right]$	Angular position vector	rad
$\left[\begin{array}{ccc} u & v & w \end{array}\right]$	Linear Velocity vector	m/s
$\left[\begin{array}{ccc} p & q & r \end{array}\right]$	Angular velocity vector	rad/s
$\left[\begin{array}{ccc} I_{xx} & I_{yy} & I_{zz} \end{array}\right]$	Moment of inertia vector	$kg\cdot m^{2}$
*F*_*th*_	Total thrust generated by rotors	N
$\left[\begin{array}{ccc} \tau_{x} & \tau_{y} & \tau_{z} \end{array}\right]$	Control torques	N.m
*g*	Gravitational force	$m/s^{2}$
*m*	Total mass	kg
$\left[\begin{array}{cccc} \omega_{1} & \omega_{2} & \omega_{3} & \omega_{4} \end{array}\right]$	Rotors speeds vector	rad/s
*b*	Thrust coefficient	N.s $s^{2}$
*l*	Motor to center length	m
*w*_*r*_	residual rotor speed	rad/s
*d*	Drag coefficient	N.m.s.s
*Jr*	rotor inertia vector	$kg\cdot m^{2}$

**External forces and moments**

### Force

The force exerted on a quadcopter influences its translational motion [39]. According to Newton’s second law, this force is expressed as:

$$\sum F=F_{\text{th}}+F_{\text{grav}}=m\vec{a}$$ (10)

In the body-fixed reference frame, translational dynamics are influenced by two main forces:

#### Thrust force.

The thrust force is the combined upward thrust generated by the propellers. This force is quadratically related to the angular velocities of the rotors as $F_{i}=b\omega_{i}^{2}$ [39]. The total thrust in the body frame is given by:

$$F_{\text{th}}^{B}=F_{1}+F_{2}+F_{3}+F_{4}=b\left(\omega_{1}^{2}+\omega_{2}^{2}+\omega_{3}^{2}+\omega_{4}^{2}\right)$$ (11)

In the Earth’s inertial reference frame, the thrust force can be expressed using the rotation matrix:

$$F_{\text{th}}^{E}={\;\;}_{B}^{E}R\times F_{\text{th}}^{B}$$ (12)

where

$$F_{\text{th}}^{E}=[\begin{array}{c} (\cos(\phi )\sin(\theta )\cos(\psi )+\sin(\phi )\sin(\psi ))F_{\text{th}}^{B}\\ (\cos(\phi )\sin(\theta )\sin(\psi )-\sin(\phi )\cos(\psi ))F_{\text{th}}^{B}\\ (\cos(\phi )\cos(\theta ))F_{\text{th}}^{B} \end{array}]$$ (13)

#### Gravitational force.

The gravitational force, in the Earth’s inertial reference frame, is expressed as:

$$F_{\text{grav}}^{E}=[\begin{array}{c} 0\\ 0\\ -mg \end{array}]$$ (14)

#### Net force.

In the Earth’s inertial frame, the net force is given by:

$$\sum F^{E}=F_{\text{th}}^{E}+F_{\text{grav}}^{E}=m\vec{a}$$ (15)

Let:


$$U_{1}=F_{\text{th}}^{B}=b\left(\omega_{1}^{2}+\omega_{2}^{2}+\omega_{3}^{2}+\omega_{4}^{2}\right)$$

From the above, the translational dynamics in the Earth’s inertial reference frame are:

$$\left\{ \begin{array}{c} \ddot{x}=\left(\cos(\phi )\sin(\theta )\cos(\psi )+\sin(\phi )\sin(\psi )\right)\frac{U_{1}}{m}\\ \ddot{y}=\left(\cos(\phi )\sin(\theta )\sin(\psi )-\sin(\phi )\cos(\psi )\right)\frac{U_{1}}{m}\\ \ddot{z}=\left(\cos(\phi )\cos(\theta )\right)\frac{U_{1}}{m}-g \end{array}\right.$$ (16)

### Rotational dynamics

The rotational dynamics, accounting for net torques and reaction forces, are described as:

$$\sum \tau =M_{\text{thrust}}+F_{\text{gyro}}$$ (17)

These dynamics can be determined using both the body-fixed and Earth inertial frames.

**Moments of thrust forces**

For this paper, we assume that clockwise (CW) propeller rotation is positive, and counterclockwise (CCW) rotation is negative [39]. The moments of thrust forces in the body frame are given by:

$$\left\{ \begin{array}{c} \tau_{x}^{B}=\frac{\sqrt{2}}{2}lb\left(\omega_{1}^{2}+\omega_{2}^{2}-\omega_{3}^{2}-\omega_{4}^{2}\right)\\ \tau_{y}^{B}=\frac{\sqrt{2}}{2}lb\left(\omega_{1}^{2}-\omega_{2}^{2}-\omega_{3}^{2}+\omega_{4}^{2}\right)\\ \tau_{z}^{B}=-d\omega_{1}^{2}+d\omega_{2}^{2}-d\omega_{3}^{2}+d\omega_{4}^{2} \end{array}\right.$$ (18)

From Eq (18), let $\tau_{x}^{B}=U_{2}$ (roll), $\tau_{y}^{B}=U_{3}$ (pitch), and $\tau_{z}^{B}=U_{4}$ (yaw). Balance is achieved when the total rotor speeds sum to zero, ensuring stability, as propellers 2 and 4 rotate CW and propellers 1 and 3 rotate CCW. Otherwise, imbalance causes gyroscopic effects that influence roll and pitch rates.

The gyroscopic force in the body frame is:

$$F_{\text{gyro}}^{B}=Jr[\begin{array}{c} qw_{r}\\ -pw_{r}\\ 0 \end{array}]$$ (19)

where $w_{r}=\omega_{1}-\omega_{2}+\omega_{3}-\omega_{4}$ and *J* is the moment of inertia.

In the inertial frame, the rate of change of angular momentum $\vec{L}$ equals the total torques acting on the quadrotor. Since the geometry is symmetric, the inertia tensor is represented as:


$$J=[\begin{array}{ccc} I_{xx} & 0 & 0\\ 0 & I_{yy} & 0\\ 0 & 0 & I_{zz} \end{array}]$$

The total torque in the body frame is:

$$\sum M^{B}=M_{\text{thrust}}^{B}+F_{\text{gyro}}^{B}=\dot{\vec{L}}+\vec{\omega }\times \vec{L}$$ (20)

The angular acceleration in the body frame is expressed as:

$$\left\{ \begin{array}{c} \dot{p}=\frac{U_{2}}{I_{xx}}-\frac{qr(I_{yy}-I_{zz})}{I_{xx}}+\frac{Jrw_{r}q}{I_{xx}}\\ \dot{q}=\frac{U_{3}}{I_{yy}}-\frac{rp(I_{zz}-I_{xx})}{I_{yy}}-\frac{Jrw_{r}p}{I_{yy}}\\ \dot{r}=\frac{U_{4}}{I_{zz}}-\frac{pq(I_{xx}-I_{yy})}{I_{zz}} \end{array}\right.$$ (21)

The quadrotor’s dynamics are described by four control inputs, represented as:


$$𝐔={\left[\begin{array}{cccc} U_{1} & U_{2} & U_{3} & U_{4} \end{array}\right]}^{T},$$

where:

- *U*_1_ represents the total thrust force (throttle) applied to the quadrotor body,
- *U*_2_ is the torque affecting the roll angle,
- *U*_3_ is the torque affecting the pitch angle, and
- *U*_4_ is the torque affecting the yaw (heading) angle.

The quadrotor’s translational and rotational dynamics are governed by the following equations [39]:

$$\left\{ \begin{array}{l} \ddot{x}=\left(\cos(\phi )\sin(\theta )\cos(\psi )+\sin(\phi )\sin(\psi )\right)\frac{U_{1}}{m}\;\ddot{y}=\left(\cos(\phi )\sin(\theta )\sin(\psi )-\sin(\phi )\cos(\psi )\right)\frac{U_{1}}{m}\;\ddot{z}=\left(\cos(\phi )\cos(\theta )\right)\frac{U_{1}}{m}-g\;\ddot{\phi }=\frac{U_{2}}{I_{xx}}-\frac{\left(I_{xx}-I_{yy}\right)}{I_{xx}}\dot{\theta }\dot{\psi }+\frac{J_{r}\dot{\theta }w_{r}}{I_{xx}}\;\ddot{\theta }=\frac{U_{3}}{I_{yy}}-\frac{\left(I_{xx}-I_{zz}\right)}{I_{yy}}\dot{\phi }\dot{\psi }-\frac{J_{r}\dot{\phi }w_{r}}{I_{yy}}\;\ddot{\psi }=\frac{U_{4}}{I_{zz}}-\frac{\left(I_{yy}-I_{xx}\right)}{I_{zz}}\dot{\phi }\dot{\theta } \end{array}\right.$$ (22)

The desired roll $\left(\phi_{r}\right)$ and pitch $\left(\theta_{r}\right)$ angles are derived from Eq (22) as:

$$\left\{ \begin{array}{c} \phi_{r}=\tan^{-1}\left(\frac{\cos(\theta_{r})}{\ddot{z}+g}(\ddot{x}\sin(\psi )-\ddot{y}\cos(\psi ))\right)\\ \theta_{r}=\tan^{-1}\left(\frac{1}{\ddot{z}+g}(\ddot{x}\cos(\psi )+\ddot{y}\sin(\psi ))\right) \end{array}\right.$$ (23)

The control inputs are used to calculate the rotor angular velocities, with their inverse relationship given as:

$$\left\{ \begin{array}{c} \omega_{1}=\sqrt{\frac{U_{1}}{4b}+\sqrt{2}\frac{U_{2}}{4bl}+\sqrt{2}\frac{U_{3}}{4bl}+\frac{U_{4}}{4d}}\\ \omega_{2}=\sqrt{\frac{U_{1}}{4b}+\sqrt{2}\frac{U_{2}}{4bl}-\sqrt{2}\frac{U_{3}}{4bl}-\frac{U_{4}}{4d}}\\ \omega_{3}=\sqrt{\frac{U_{1}}{4b}-\sqrt{2}\frac{U_{2}}{4bl}-\sqrt{2}\frac{U_{3}}{4bl}+\frac{U_{4}}{4d}}\\ \omega_{4}=\sqrt{\frac{U_{1}}{4b}-\sqrt{2}\frac{U_{2}}{4bl}+\sqrt{2}\frac{U_{3}}{4bl}-\frac{U_{4}}{4d}} \end{array}\right.$$ (24)

### Virtual control

The quadrotor is underactuated, with 4 control inputs and 6 states to be controlled. The states are indirectly controlled through the translational dynamics [34–36,39]:

$$\left\{ \begin{array}{cc} V_{x}=\ddot{x} & =(\cos(\phi )\sin(\theta )\cos(\psi )+\sin(\phi )\sin(\psi ))\frac{U_{1}}{m}\\ V_{y}=\ddot{y} & =(\cos(\phi )\sin(\theta )\sin(\psi )-\sin(\phi )\cos(\psi ))\frac{U_{1}}{m}\\ V_{z}=\ddot{z} & =(\cos(\phi )\cos(\theta ))\frac{U_{1}}{m}-g \end{array}\right.$$ (25)

where $V_{x}$, $V_{y}$, and $V_{z}$ are the virtual control inputs.

Based on these equations, the lift force (altitude control signal) and the desired Euler angles are calculated as:

$$\left\{ \begin{array}{cc} U_{1} & =m\sqrt{V_{x}^{2}+V_{y}^{2}+{\left(V_{z}+g\right)}^{2}}\\ \theta_{r} & =\arctan\left(\frac{V_{x}\cos(\psi_{r})+V_{y}\sin(\psi_{r})}{V_{z}+g}\right)\\ \phi_{r} & =\arctan\left(\frac{\cos(\theta_{r})\left(V_{x}\sin(\psi_{r})-V_{y}\cos(\psi_{r})\right)}{V_{z}+g}\right) \end{array}\right.$$ (26)

## 3 Problem statement

Unmanned Aerial Vehicles (UAVs) exhibit complex dynamics involving multiple interconnected states (*x*, *y*, *z*, *ϕ*, *θ*, *ψ*), making control system design challenging. Traditional PID controllers, though widely used, struggle to maintain robust performance due to their fixed gains. Advanced techniques, such as fuzzy logic-based PID (FPID) and neural network-based PID (NNPID), have been explored for adaptive control but face limitations when applied independently. FPID offers heuristic adaptability but lacks precision for certain dynamic states, while NNPID provides precision but is computationally intensive and less flexible in real-time scenarios. This creates a gap in achieving an optimal balance of stability, adaptability, and performance across all UAV states. Therefore, a more robust and efficient control strategy is required to address these challenges and enhance overall system efficiency.

### 3.1 Proposed solution

To overcome the limitations of standalone FPID and NNPID controllers, this research proposes a hybrid control strategy (NNPID+FPID). The hybrid approach combines the strengths of both methods, integrating neural networks and fuzzy logic to adaptively tune the PID gains (*K*_*p*_, *K*_*i*_, *K*_*d*_), thereby optimizing control for UAV dynamics.

## 4 Controller design

**Fig 3:**
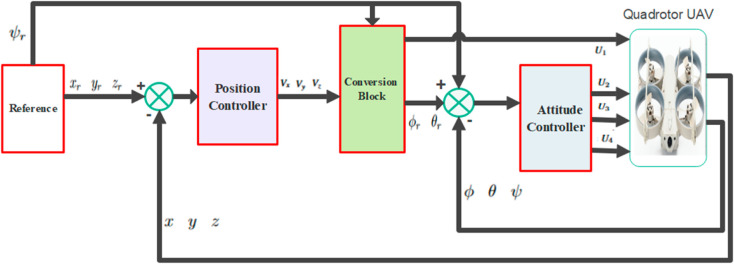
Control scheme block diagram [34].

This section presents the design of NNPID, FPID, and the hybrid NNPID+FPID controllers.The Fuzzy PID (FPID) controller integrates the traditional PID structure with fuzzy logic for adaptive gain adjustment in real-time, enhancing performance in nonlinear systems. In contrast, the Neural Network PID (NNPID) controller uses a neural network to fine-tune PID parameters for optimal control of complex systems like quadrotors. To improve trajectory tracking, the control deviation at each moment is correlated with the system’s entire past state. The generalized control scheme block diagram is shown in Fig 3.

The error and its derivative are defined as:

$$\left\{ \begin{array}{c} e(t)=r(t)-y(t)\\ de(t)=\frac{de(t)}{dt} \end{array}\right.$$ (27)

where $r={\left[\begin{array}{cccccc} \phi_{r} & \theta_{r} & \psi_{r} & x_{r} & y_{r} & z_{r} \end{array}\right]}^{T}$ is the desired reference, and $y={\left[\begin{array}{cccccc} \phi & \theta & \psi & x & y & z \end{array}\right]}^{T}$ is the quadrotor output.

### 4.1 Fuzzy PID Controller Design (FPID)

The FPID controller uses a fuzzy system for online PID gain tuning [43]. This is essential due to the nonlinear and underactuated nature of quadrotors, making classical PID control inadequate. The design involves the following steps:

1. **Define Tuning Rules:** Establish fuzzy IF-THEN rules based on system requirements to determine PID gain adjustments in response to various inputs.
2. **Create a Fuzzy System:** Combine the rules into a fuzzy system capable of real-time PID tuning based on feedback signals from the quadrotor.

**Fig 4:**
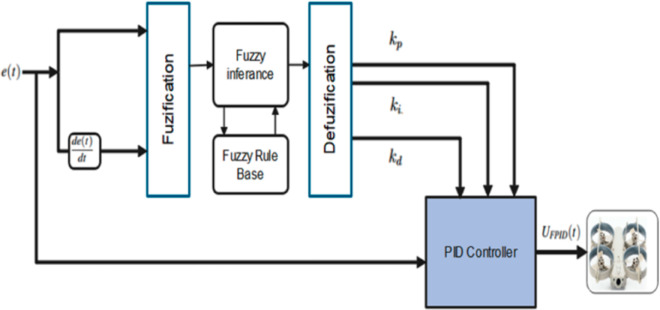
Structure of fuzzy PID controller [34].

The structure of the FPID controller is shown in Fig 4.

The fuzzy inference system uses a set of linguistic rules to determine the parameters *K*_*p*_, *K*_*i*_, and *K*_*d*_ automatically [43]. Input variables use seven fuzzy labels (NB: Negative Big, NM: Negative Medium, NS: Negative Small, ZO: Zero, PS: Positive Small, PM: Positive Medium, PB: Positive Big), while output variables use seven labels (VVS: Very Very Small, VS: Very Small, S: Small, M: Medium, B: Big, VB: Very Big, VVB: Very Very Big). These are detailed in Tables 4 and 5.

**Table 4:** Fuzzy rules for $K_{d}$ [43].

de\e	NB	NM	NS	Z	PS	PM	PB
NB	M	B	VB	VVB	VB	B	M
NM	S	M	B	VB	B	M	S
NS	VS	S	M	B	M	S	VS
Z	VVS	VS	S	M	S	VS	VVS
PS	VS	S	M	B	M	S	VS
PM	S	M	B	VB	B	M	S
PB	M	B	VB	VVB	VB	B	M

**Table 5:** Fuzzy Rules for $K_{p}$ and $K_{i}$ [43].

de\e	NB	NM	NS	Z	PS	PM	PB
NB	M	S	VS	VVS	VS	S	M
NM	B	M	S	VS	S	M	B
NS	VB	B	M	S	M	B	VB
Z	VVB	VB	B	M	B	VB	VVB
PS	VB	B	M	S	M	B	VB
PM	B	M	S	VS	S	M	B
PB	M	S	VS	VVS	VS	S	M

For position and attitude control, the error is normalized to [–1,1], the error rate to [–10,10], and the output ranges are [0.2,0.7], [0.001,0.01], and [0.1,0.15] for proportional, integral, and derivative gains, respectively. The system employs product-sum inference and center-of-gravity defuzzification [34,43–45].

The fuzzy controller output is:

$$U_{\text{FPID}}(t)=k_{p}e(t)+k_{i}\int_{0}^{t}e(t)dt+k_{d}\frac{de(t)}{dt}$$ (28)

### 4.2 Neural PID Controller (NNPID)

**Fig 5:**
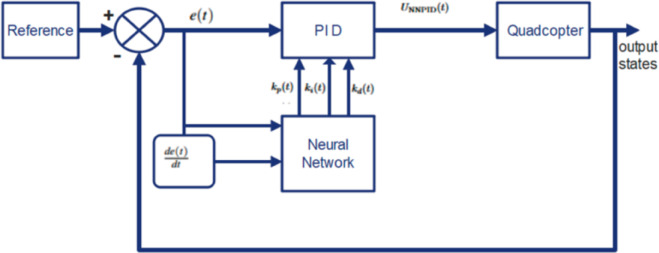
Structure of Neural Network PID (NNPID) Controller.

A Neural Network-Based Self-Tuning PID Controller integrates artificial neural networks with PID control to dynamically adjust controller parameters, enhancing robustness and adaptability to varying system dynamics and operating conditions [46]. Traditional PID tuning methods, such as the Ziegler-Nichols approach, determine fixed coefficients that lack adaptability under varying model parameters and operating conditions. To address this limitation, the NNPID controller combines the simplicity of PID control with the adaptability of artificial neural network. The structure of the NNPID controller is shown in Fig 5.

#### 4.2.1 Optimization objective.

The objective is to minimize the squared errors *e*_*p*_(*t*), *e*_*i*_(*t*), and *e*_*d*_(*t*) over time by dynamically adjusting the neural network weights and biases. The cost function is defined as:

$$J=\frac{1}{2}\left(e_{p}^{2}+e_{i}^{2}+e_{d}^{2}\right)$$ (29)

where *e*_*p*_, *e*_*i*_, and *e*_*d*_ represent the proportional, integral, and derivative errors, respectively.

#### 4.2.2 Gradient descent.

The neural network’s weights (*W*) and biases (*b*) are updated using the gradient descent optimization algorithm to minimize the cost function [47,48]. The cost function is in Eq (29): The updates for weights and biases are computed as follows:

$$W^{\left(t+1\right)}=W^{\left(t\right)}-\eta \frac{\partial J}{\partial W}$$ (30)

$$b^{\left(t+1\right)}=b^{\left(t\right)}-\eta \frac{\partial J}{\partial b}$$ (31)

where:

- ${\text{W}}^{\left(\text{t}\right)}$, ${\text{b}}^{\left(\text{t}\right)}$: Weights and biases at iteration *t* respectively.
- *η*: Learning rate, controlling the step size of the updates.
- $\frac{\partial J}{\partial W}$, $\frac{\partial J}{\partial b}$: Gradients of the cost function with respect to the weights and biases respectively.

The gradients are computed using backpropagation, which calculates the partial derivatives of the cost function with respect to each parameter in the neural network. By iteratively updating *W* and *b*, the neural network minimizes the cost function, ensuring improved control performance over time.

#### 4.2.3 Learning rate and iterations.

The optimization process is controlled by:

- **Learning rate (*η*)**: Step size for gradient descent updates.
- **Number of iterations ($N_{\text{max}}$)**: Maximum number of optimization steps.

### Error reduction

The errors *e*_*p*_(*t*), *e*_*i*_(*t*), and *e*_*d*_(*t*) are iteratively reduced using PID gains and the neural network tuned PID control is:

$$U_{\text{NNPID}}(t)=k_{p}(t)e_{p}(t)+k_{i}(t)e_{i}(t)+k_{d}(t)e_{d}(t)$$ (32)

where:

- $U_{\text{NNPID}}(t)$: Neural Network tuned adaptive PID control signal.
- *k*_*p*_(*t*): Proportional gain tuned by neural network.
- *k*_*i*_(*t*): Integral gain tuned by neural network.
- *k*_*d*_(*t*): Derivative gain tuned by neural network.

#### 4.2.4 Implementation details.

This article employs a feedforward neural network optimized via gradient descent (GD) due to its computational efficiency, requiring only first-order derivatives [49–51]. Weights and biases are initialized randomly and updated in real-time through online learning, progressively minimizing errors and improving system performance. Simulation data initialize errors based on the reference signal and deviations from the controlled plant’s output. The neural network uses:

- **Configuration**: 10 neurons.
- **Learning rate**: 0.001.
- **Maximum iterations**: 1000.
- **Stopping criterion**: Loss function reaches 10^−6^.

PID gains are initialized to zero and optimized iteratively using the neural network. The full optimization procedure is detailed in Algorithm 1.

#### 4.2.5 Algorithm for Neural PID Optimization (NNPID).

The neural network optimization process is summarized in Algorithm 1, systematically presenting all steps for real-time parameter tuning.

**Algorithm 1. Neural network for PID parameter optimization.**



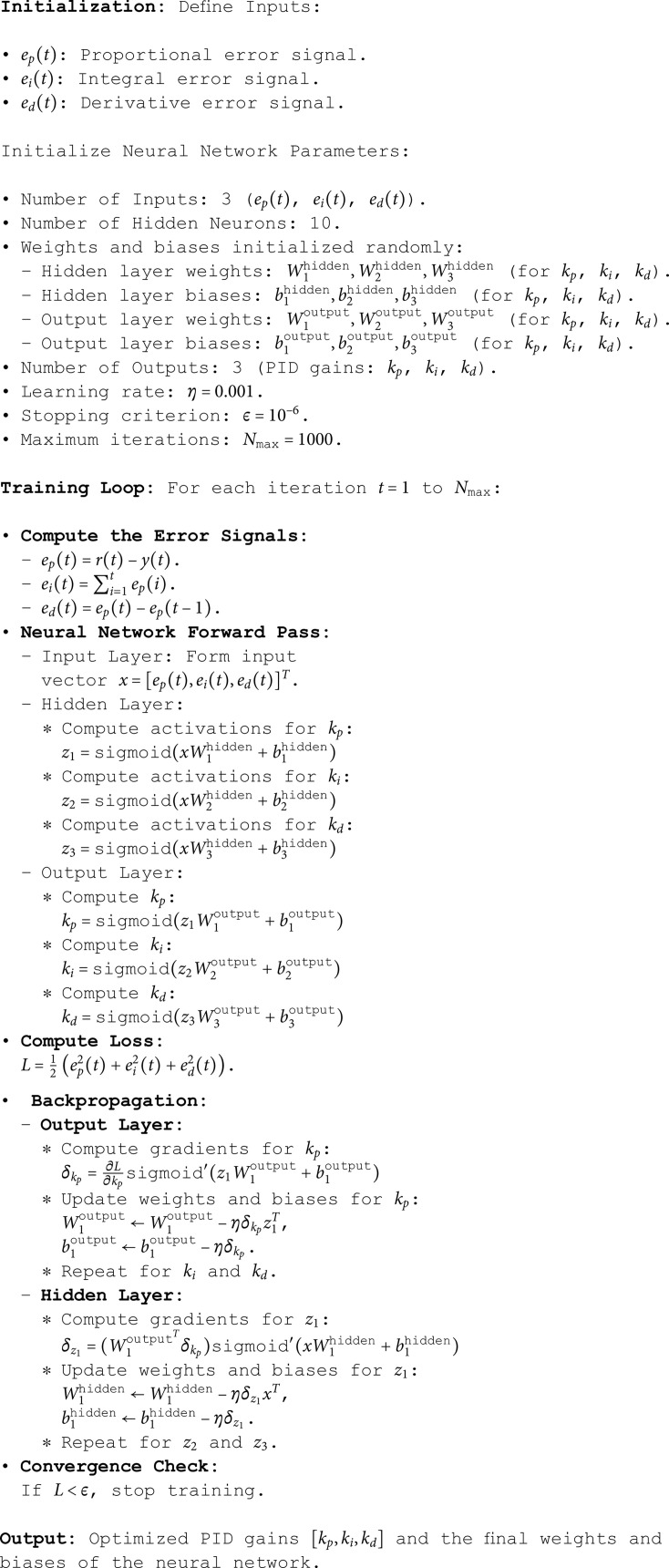



### 4.3 Hybrid Neural Fuzzy PID (NNPID+FPID) controller

This section presents the combined Hybrid Neural Network and Fuzzy Logic-based PID tuning control algorithm (NNPID+FPID). The hybrid approach is designed to address the limitations of standalone Neural Network (NNPID) and Fuzzy Logic self-tuning (FPID) controllers for stable and precise trajectory tracking of quadrotor UAVs.

**Fig 6:**
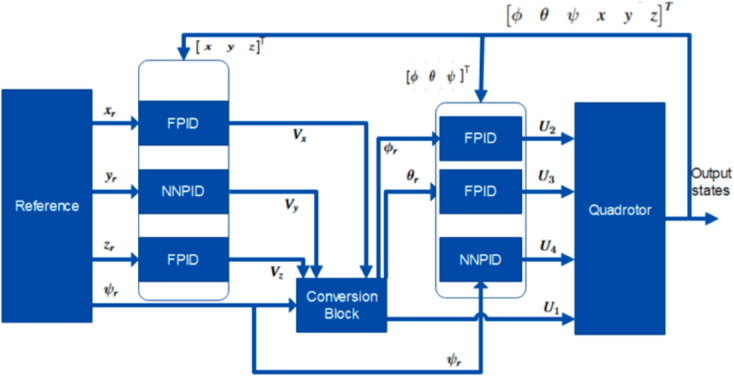
Proposed Hybrid (NNPID+FPID) Control Scheme.

The NNPID controller performs well in lateral dynamics and heading control, while the FPID controller excels in longitudinal, altitude, pitch, and roll dynamics. To leverage the strengths of both approaches, the proposed hybrid control scheme integrates NNPID and FPID for enhanced trajectory tracking. The hybrid control architecture is illustrated in Fig 6.

### 4.4 Lyapunov stability analysis for fuzzy PID-controlled altitude dynamics

To analyze the stability of the fuzzy PID controller applied to the altitude (*z*-axis) of the quadrotor UAV, we consider the simplified translational dynamics along the vertical axis. From the original model described in the Eq (22):

$$\ddot{z}=\left(\cos(\phi )\cos(\theta )\right)\frac{U_{1}}{m}-g$$ (33)

Assuming small angle approximations around hover (cos(ϕ)cos(θ)≈1), the altitude dynamics reduce to:

$$\ddot{z}=\frac{U_{1}}{m}-g$$ (34)

Let $u=\frac{U_{1}}{m}$, then:

$$\ddot{z}=u-g$$ (35)

The control input *u* is generated by a fuzzy-tuned PID controller:

$$u(t)=k_{p}e(t)+k_{i}\int_{0}^{t}e(\tau )\;d\tau +k_{d}\dot{e}(t)$$ (36)

where *e*(*t*) = *z*_*r*_(*t*)−*z*(*t*) is the altitude error, and the gains $k_{p},k_{i},k_{d}$ are adaptively determined using fuzzy inference based on error *e* and error derivative $\dot{e}$. The gain ranges are constrained to:

$$k_{p}\in [0.2,0.7]$$

$$k_{i}\in [0.001,0.01]$$

$$k_{d}\in [0.1,0.15]$$

#### State-space formulation.

Define the following states:

$$x_{1}=e=z_{r}-z$$ (37)

$$x_{2}=\dot{e}={\dot{z}}_{r}-\dot{z}$$ (38)

$$x_{3}=\int_{0}^{t}e(\tau )\;d\tau$$ (39)

Assuming *z*_*r*_ is constant (${\dot{z}}_{r}=0$), the state-space model becomes:

$${\dot{x}}_{1}=x_{2}$$ (40)

$${\dot{x}}_{2}=g-(k_{p}x_{1}+k_{i}x_{3}+k_{d}x_{2})$$ (41)

$${\dot{x}}_{3}=x_{1}$$ (42)

#### Lyapunov candidate function.

We select a Lyapunov candidate function that includes the error, its rate, and integral:

$$V(x)=\frac{1}{2}x_{1}^{2}+\frac{1}{2}x_{2}^{2}+\frac{1}{2}k_{i}x_{3}^{2}$$ (43)

This function is positive definite. The time derivative is:

$$\dot{V}=x_{1}{\dot{x}}_{1}+x_{2}{\dot{x}}_{2}+k_{i}x_{3}{\dot{x}}_{3}$$ (44)

$$=x_{1}x_{2}+x_{2}(g-k_{p}x_{1}-k_{i}x_{3}-k_{d}x_{2})+k_{i}x_{3}x_{1}$$ (45)

$$=(1-k_{p})x_{1}x_{2}+gx_{2}-k_{i}x_{3}x_{2}-k_{d}x_{2}^{2}+k_{i}x_{3}x_{1}$$ (46)

#### Bounding cross terms.

To ensure $\dot{V}\leq 0$, we apply Young’s inequality to the cross terms:

$$(1-k_{p})x_{1}x_{2}\leq \frac{(1-k_{p}{)}^{2}}{2\epsilon_{1}}x_{1}^{2}+\frac{\epsilon_{1}}{2}x_{2}^{2}$$ (47)

$$gx_{2}\leq \frac{g^{2}}{2\epsilon_{2}}+\frac{\epsilon_{2}}{2}x_{2}^{2}$$ (48)

$$-k_{i}x_{3}x_{2}\leq \frac{k_{i}^{2}}{2\epsilon_{3}}x_{3}^{2}+\frac{\epsilon_{3}}{2}x_{2}^{2}$$ (49)

$$k_{i}x_{3}x_{1}\leq \frac{k_{i}^{2}}{2\epsilon_{4}}x_{3}^{2}+\frac{\epsilon_{4}}{2}x_{1}^{2}$$ (50)

Substituting these bounds into $\dot{V}$, we obtain:

$$\dot{V}\leq -k_{d}x_{2}^{2}+\left(\frac{(1-k_{p}{)}^{2}}{2\epsilon_{1}}+\frac{\epsilon_{4}}{2}\right)x_{1}^{2}+\left(\frac{\epsilon_{1}+\epsilon_{2}+\epsilon_{3}}{2}\right)x_{2}^{2}\;\;+\left(\frac{k_{i}^{2}}{2\epsilon_{3}}+\frac{k_{i}^{2}}{2\epsilon_{4}}\right)x_{3}^{2}+\frac{g^{2}}{2\epsilon_{2}}$$ (51)

#### Stability conclusion.

By choosing:

- $k_{p}\approx 1$ to minimize $(1-k_{p}){\;\;}^{2}$,
- sufficiently large *k*_*d*_ to dominate terms in $x_{2}^{2}$,
- small *k*_*i*_ (within its bounded fuzzy range),

we can ensure that all the terms in $\dot{V}$ are either negative or bounded. Therefore, $\dot{V}\leq 0$ outside a small residual set, ensuring that the system is uniformly ultimately bounded.

The fuzzy PID controller, by adaptively selecting gains according to the error dynamics via rule tables, maintains boundedness and convergence of the altitude tracking error. Hence, the closed-loop system is Lyapunov stable under fuzzy PID control.

## 5 Simulation

The quadrotor simulation integrates the parameters in Table 6 into the MATLAB environment. The proposed NNPID+FPID controller’s performance is evaluated for trajectory tracking and benchmarked against standard FPID and NNPID controllers under varying conditions.

**Table 6:** Parameter values [34].

Parameter	Value
*I*_*xx*_	$8.5532\times 10^{-3}$
*I*_*yy*_	$8.5532\times 10^{-3}$
*I*_*zz*_	$1.476\times 10^{-2}$
*g*	$9.81\times 10^{0}$
*m*	$1\times 10^{0}$
*b*	$7.66\times 10^{-5}$
*d*	$5.63\times 10^{-6}$
*l*	$2.2\times 10^{-1}$
*J*_*r*_	$0.1\times 10^{-3}$

Simulations are conducted using MATLAB 2023a Simulink, and results confirm the superior performance of the hybrid NNPID+FPID controller for helical trajectory tracking. The reference signal for helical trajectory tracking is defined as:

$$\left\{ \begin{array}{c} x_{r}(t)=\cos(1.05t)\\ y_{r}(t)=\sin(1.05t)\\ z_{r}(t)=3+\frac{t}{4}\\ \psi_{r}(t)=\frac{\pi }{4} \end{array}\right.$$ (52)

**Simulation Time: *t* = 2000 seconds**

**Fig 7:**
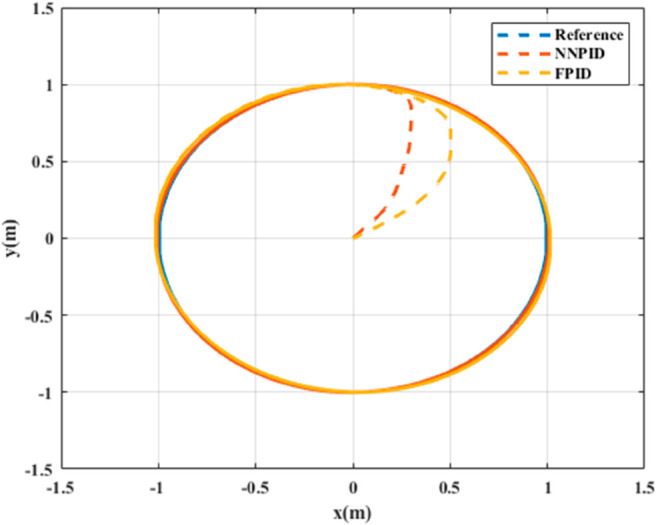
xy Tracking response for helical trajectory tracking.

**Fig 8:**
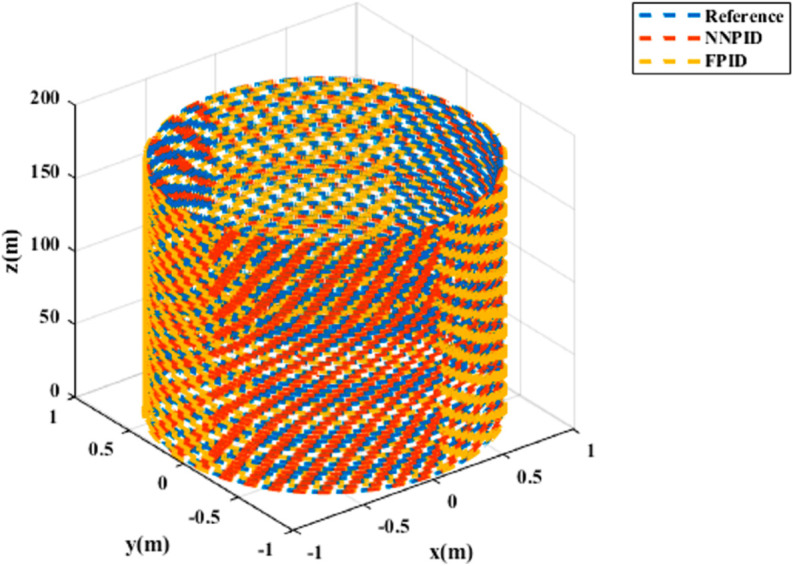
3D Plot for helical trajectory tracking.

The simulation assumes over 30 minutes of flight time to evaluate the controllers’ (NNPID and FPID) tracking performance under undisturbed conditions. The results for helical trajectory tracking are shown in Figs 7 and 8.

**Fig 9:**
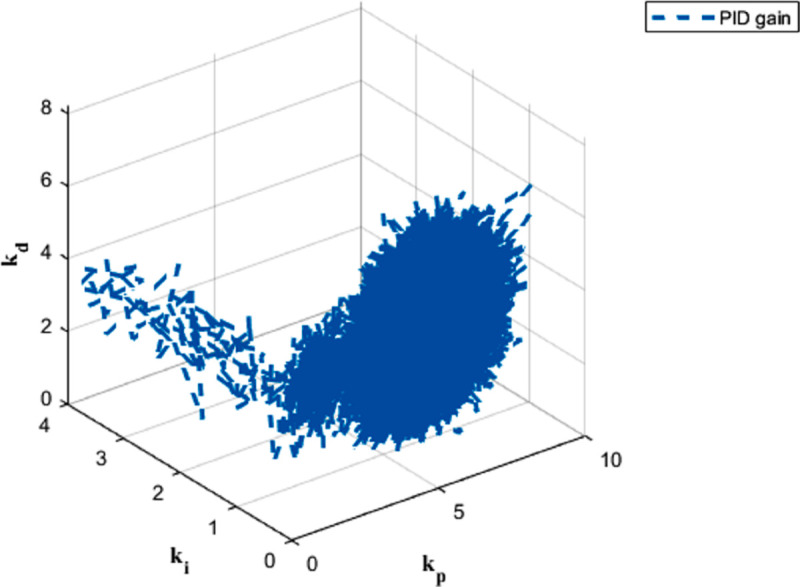
PID gain plot for x dynamics.

**Fig 10:**
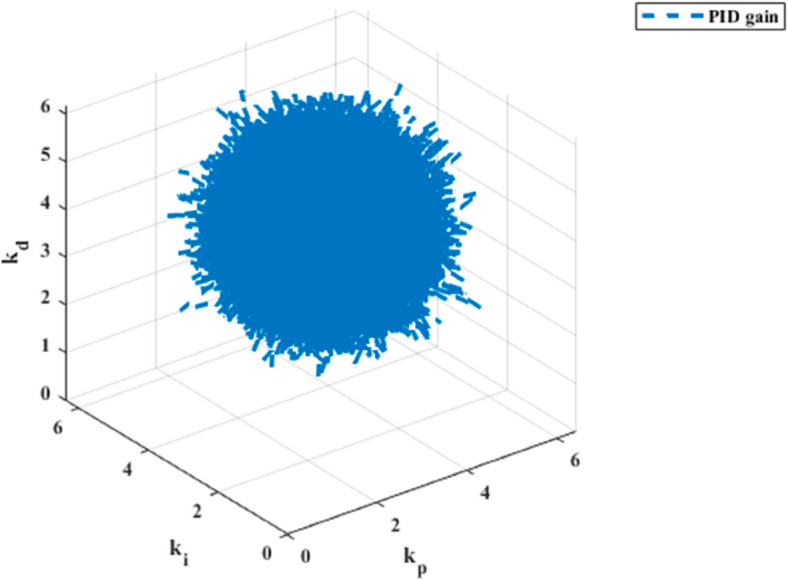
PID gain plot for y dynamics.

**Fig 11:**
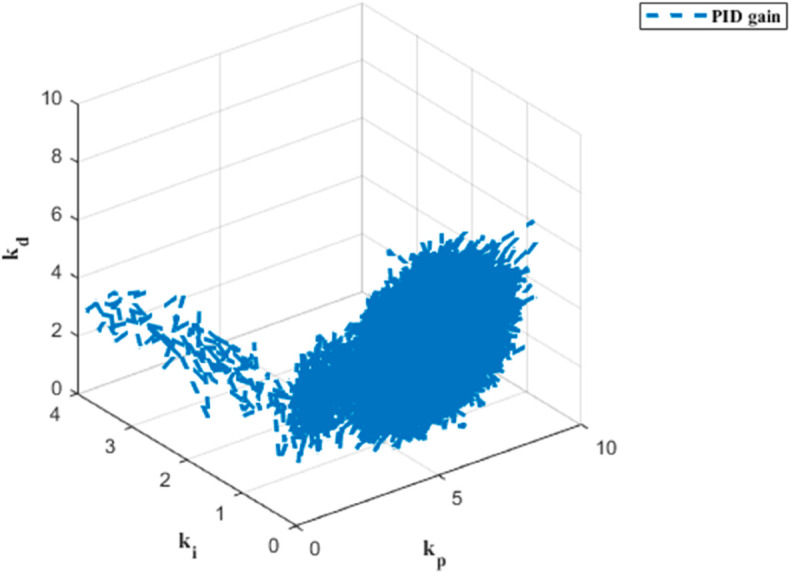
PID gain plot for z dynamics.

**Fig 12:**
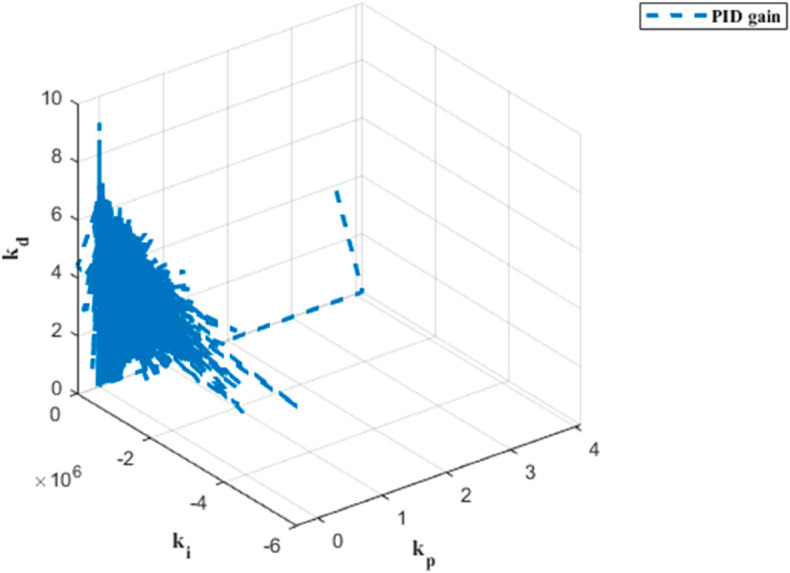
PID gain plot for *ϕ* dynamics.

**Fig 13:**
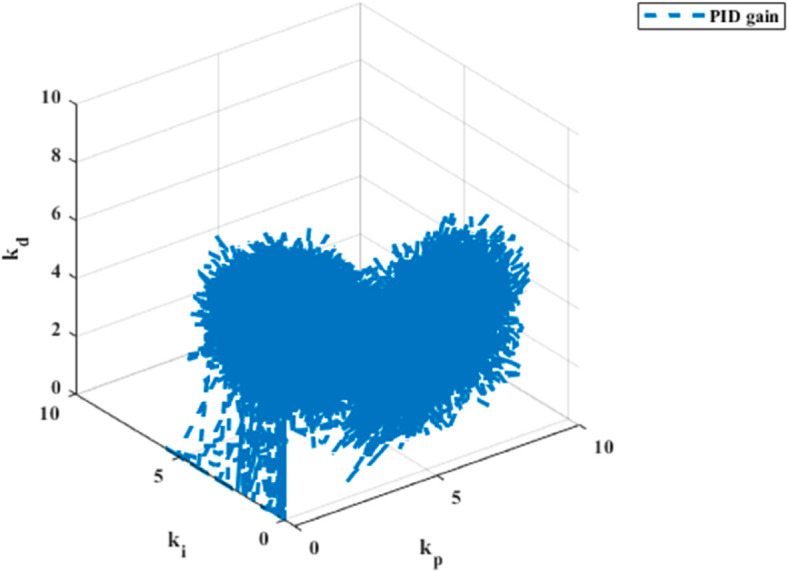
PID gain plot for *θ* dynamics.

**Fig 14:**
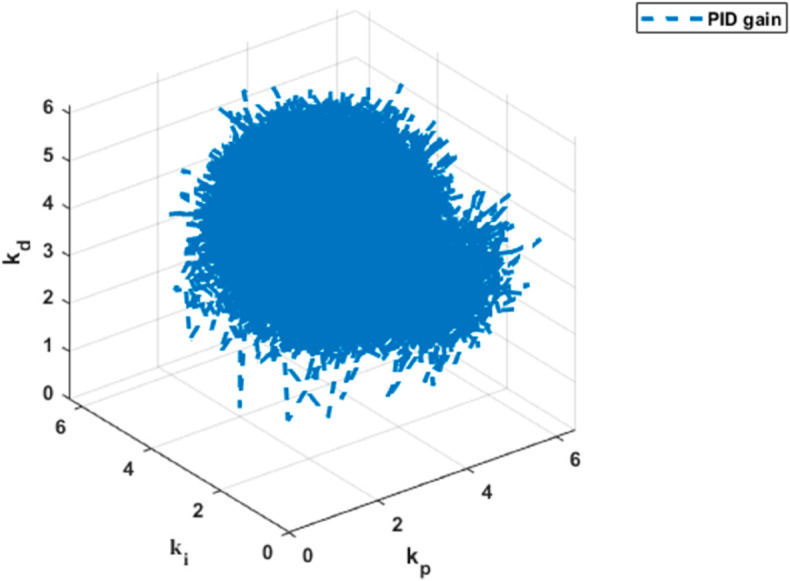
PID gain plot for *ψ* dynamics.

The PID gain results for trajectory tracking across six dynamics are depicted in Figs 9, 10, 11, 12, 13 and 14.

**Fig 15:**
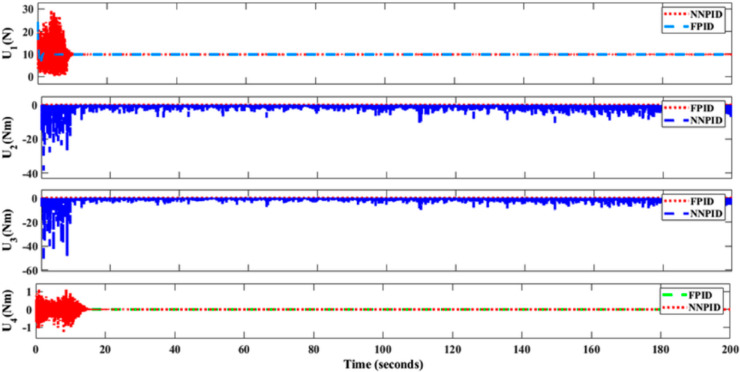
Control effort for helical trajectory tracking with standalone controllers.

**Fig 16:**
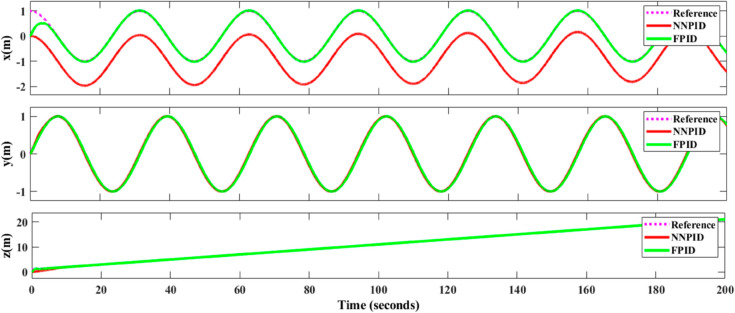
Position tracking response for helical trajectory tracking with standalone controllers.

**Fig 17:**
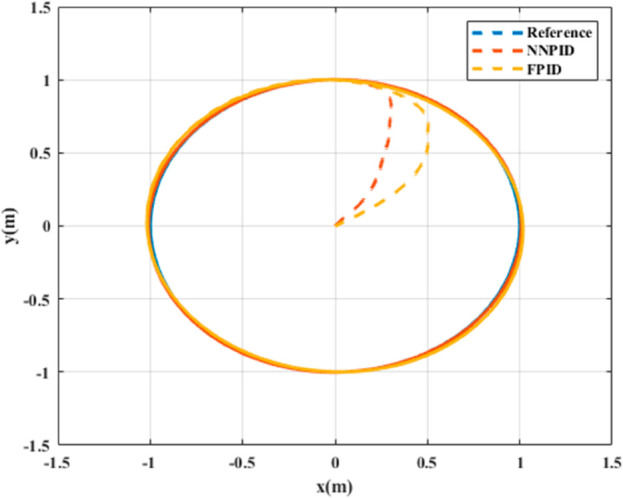
xy tracking response for helical trajectory tracking with standalone controllers.

**Fig 18:**
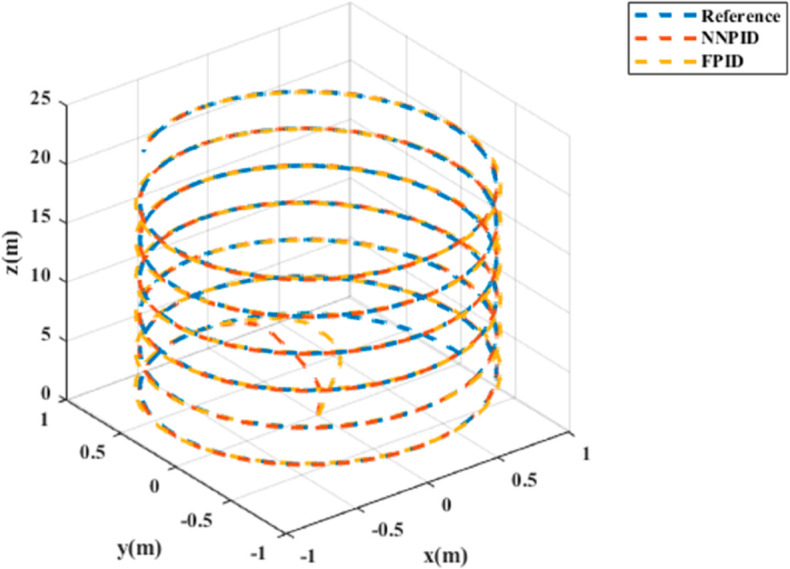
3D Plot for helical trajectory tracking with standalone controllers.

**For simulation time *t* = 200 seconds** The simulation results in undisturbed conditions for helical trajectory tracking are depicted in Figs 15, 16, 17, and 18.

## 6 Disturbance rejection capability and robustness to parameter variation of NNPID and FPID alone control

This section assesses the disturbance rejection capability and robustness of check with the NNPID and FPID controllers under conditions of parameter variation. To replicate real-world scenarios, a random force representing wind disturbances was introduced as an external input, affecting the *x*, *y*, and *z* axes of the system. Additionally, parameter variations were modeled by increasing the total quadrotor mass by 25% and the inertia values along the *x*, *y*, and *z* axes by 10%.

The applied disturbance force was set to 1 N in the *x*, *y*, and *z* directions during specific time intervals: 7–10 seconds for the *x* axis, 12–15 seconds for the *y* axis, and 17–20 seconds for the *z* axis. These conditions were designed to rigorously evaluate the system’s performance under external disturbances and dynamic parameter changes.

**Fig 19:**
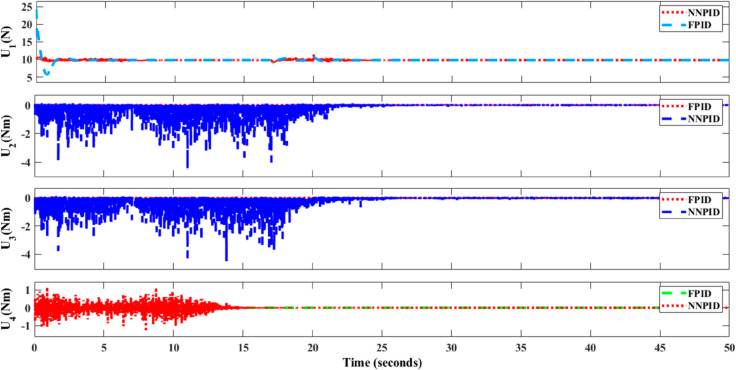
Control effort for helical trajectory tracking in the presence of input disturbances with standalone controllers.

**Fig 20:**
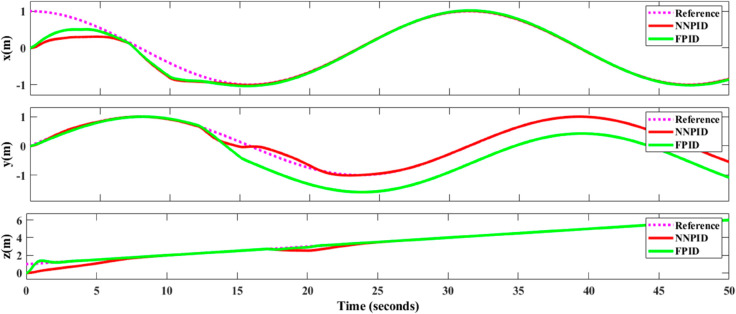
Position tracking response for helical trajectory tracking in the presence of input disturbances with standalone controllers.

**Fig 21:**
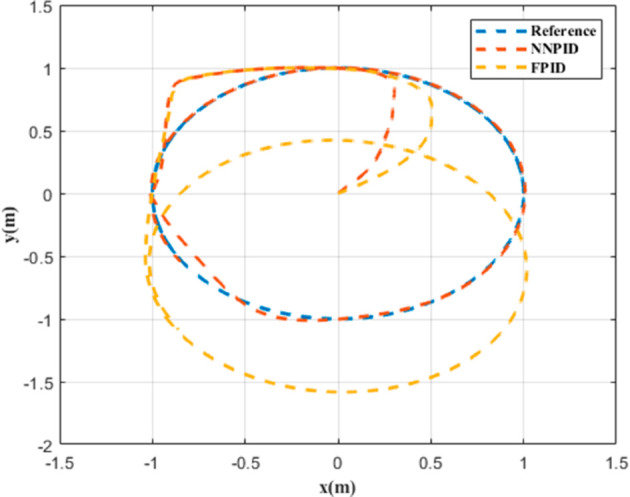
xy tracking response for helical trajectory tracking in the presence of input disturbances with standalone controllers.

**Fig 22:**
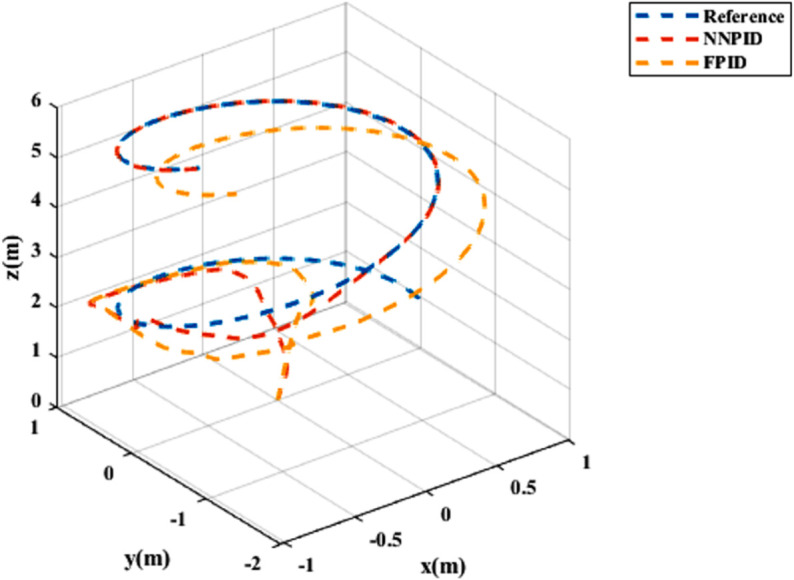
3D Plot for helical trajectory tracking in the presence of input disturbances with standalone controllers.

The simulation results for helical trajectory, incorporating the effects of the input disturbances are presented in Figs 19, 20, 21, and 22.

### 6.1 Comparison of controllers for helical trajectory based on performance criteria for NNPID and FPID alone control

This study undertakes a comprehensive comparison of two controllers— Fuzzy Proportional-Integral-Derivative (FPID), and Neural network based PID controller(NNPID) based on performance criteria in the tabular form described in Tables 7 and 8 in undisturbed conditions and disturbed (with input disturbance added) respectively.

**Table 7:** Comparison of Controllers Using mean squared error (MSE) Criteria for helical Trajectory (undisturbed condition).

States	NNPID (MSE)	FPID (MSE)
*x*	5.51	3.041
*y*	$4.878\;\times \;10^{-5}$	0.003699
*z*	8.81	0.01709
*ϕ*	$4.818\;\times \;10^{4}$	$8.96\;\times \;10^{-6}$
*θ*	1488	0.0007452
*ψ*	$2.315\;\times \;10^{-35}$	0.003558

**Table 8:** Comparison of Controllers Using Mean Squared Error (MSE) Criteria for Helical Tracking in the Presence of Input Disturbances.

States	NNPID (MSE)	FPID (MSE)
*x*	11.7	7.881
*y*	$1.07\;\times \;10^{-5}$	219.6
*z*	18.63	0.1132
*ϕ*	2528	$1.766\;\times \;10^{-6}$
*θ*	2447	0.001278
*ψ*	$2.01\;\times \;10^{-19}$	0.003558

**Discussion of strengths and limitations**

Based on the comparison of mean squared error (MSE) criteria in both undisturbed and disturbed conditions, the strengths and limitations of the Neural Network PID (NNPID) and Fuzzy Logic PID (FPID) controllers can be analyzed as follows:

#### 6.1.1 Neural Network PID (NNPID)

##### Strengths

- **Precision in *y* and *ψ* states:**
  – The NNPID demonstrates extremely low MSE values for *y* ($4.878\;\times \;10^{-5}$) and *ψ* ($2.315\;\times \;10^{-35}$) in undisturbed conditions, indicating exceptional tracking accuracy.
  – This precision holds even under disturbances, with *y* ($1.07\;\times \;10^{-5}$) and *ψ* ($2.01\;\times \;10^{-19}$) maintaining near-perfect performance.
- **Adaptability in non-linear dynamics:**
  – Neural networks are well-suited for handling the non-linear dynamics of UAVs, particularly in states requiring high trajectory accuracy, such as *y* and *ψ*.
- **Low sensitivity to disturbances in specific states:**
  – NNPID remains resilient in *y* and *ψ* states, even under disturbances.

##### Limitations

- **Poor performance in other states (x,z,ϕ,θ):**
  – In undisturbed conditions, MSE for *x* (5.51), *z* (8.81), *ϕ* ($4.818\;\times \;10^{4}$), and *θ* (1488) is significantly higher compared to FPID.
  – The performance deteriorates further under disturbances, especially for *ϕ* (2528) and *θ* (2447).
- **High computational cost:**
  – Neural networks require continuous online training, which increases computational complexity and may be less efficient in real-time control for states like x,z,ϕ,θ.

#### 6.1.2 Fuzzy Logic PID (FPID)

##### Strengths

- **Robust performance in x,z,ϕ,θ states:**
  – FPID consistently outperforms NNPID in *x*, *z*, *ϕ*, and *θ* under both undisturbed and disturbed conditions.
  – For example, in undisturbed conditions, FPID achieves much lower MSE for *x* (3.041 vs. 5.51) and *z* (0.01709 vs. 8.81). Similarly, for *ϕ* ($8.96\;\times \;10^{-6}$) and *θ* (0.0007452), FPID outperforms NNPID by orders of magnitude.
- **Resilience to disturbances in x,z,ϕ,θ:**
  – In the presence of disturbances, FPID remains effective in these states. For instance, *z* (0.1132) and *ϕ* ($1.766\;\times \;10^{-6}$) continue to outperform NNPID (18.63 and 2528, respectively).
- **Simpler and faster implementation:**
  – FPID relies on heuristic rules and membership functions, which are computationally less demanding than neural networks, making it more suitable for real-time control in x,z,ϕ,θ states.

##### Limitations

- **Lower precision in *y* and *ψ* states:**
  – FPID has higher MSE for *y* (0.003699 in undisturbed, 219.6 in disturbed conditions) and *ψ* (0.003558), highlighting its inability to match the tracking precision of NNPID in these states.
- **Limited adaptability for complex dynamics:**
  – Fuzzy logic struggles with precision in non-linear dynamics such as *y* and *ψ*, where the relationship between inputs and outputs is more intricate and requires fine-tuning that heuristic rules cannot easily provide.

## 7 Simulation result including hybrid control approach

**Fig 23:**
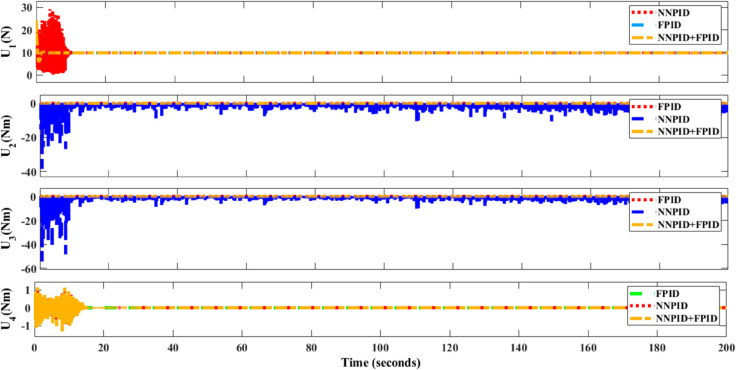
Control effort for helical trajectory tracking including hybrid controller.

**Fig 24:**
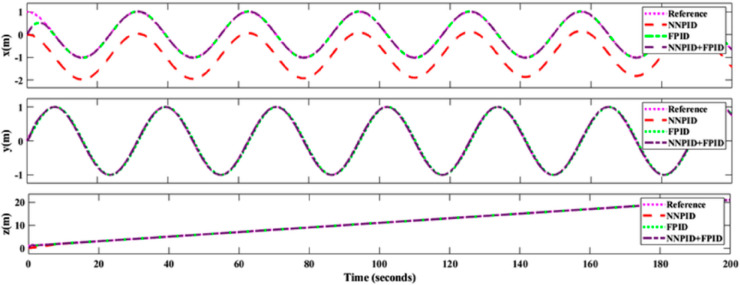
Position tracking response for helical trajectory tracking including hybrid controller.

**Fig 25:**
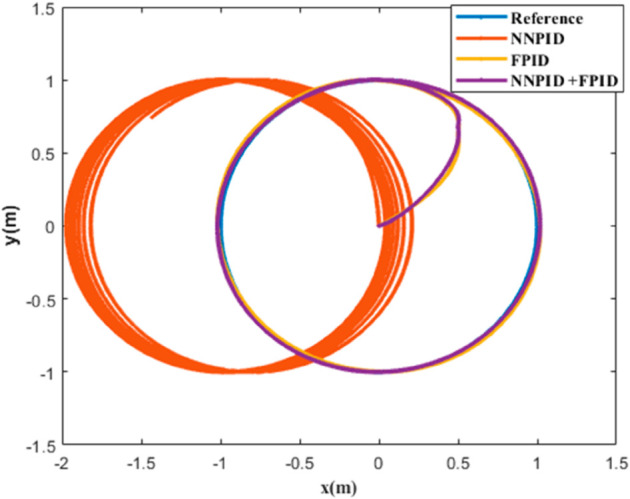
xy tracking response for helical trajectory tracking including hybrid controller.

**Fig 26:**
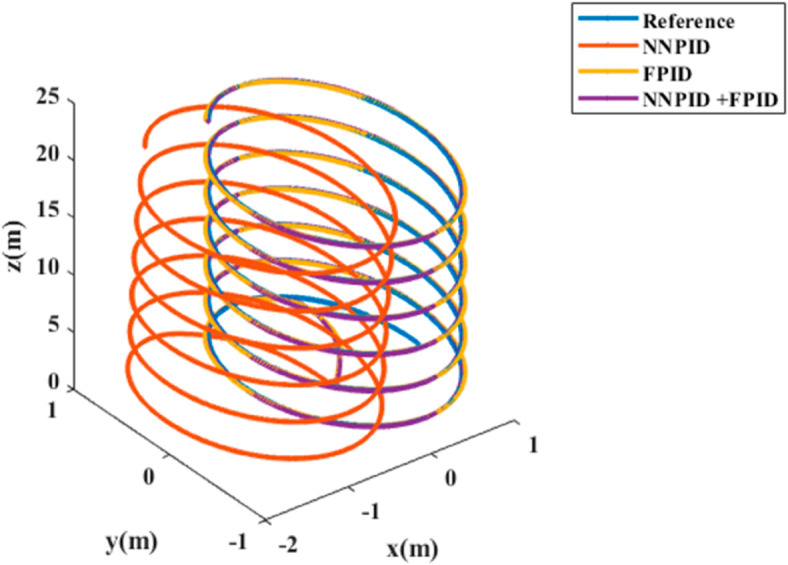
3D Plot for helical trajectory tracking including hybrid controller.

The simulation results in undisturbed conditions for helical trajectory tracking including the hybrid approach are depicted in Figs 23, 24, 25, and 26.

## 8 Disturbance rejection capability and robustness to parameter variation of NNPID+FPID, NNPID and FPID controllers

This section assesses the disturbance rejection capability and robustness of the proposed hybrid controller (NNPID+FPID) in comparison with the NNPID and FPID controllers under conditions of parameter variation. To replicate real-world scenarios, a random force representing wind disturbances was introduced as an external input, affecting the *x*, *y*, and *z* axes of the system. Additionally, parameter variations were modeled by increasing the total quadrotor mass by 25% and the inertia values along the *x*, *y*, and *z* axes by 10%.

The applied disturbance force was set to 1 N in the *x*, *y*, and *z* directions during specific time intervals: 7–10 seconds for the *x* axis, 12–15 seconds for the *y* axis, and 17–20 seconds for the *z* axis. These conditions were designed to rigorously evaluate the system’s performance under external disturbances and dynamic parameter changes.

### 8.1 Disturbance rejection capability

**Fig 27:**
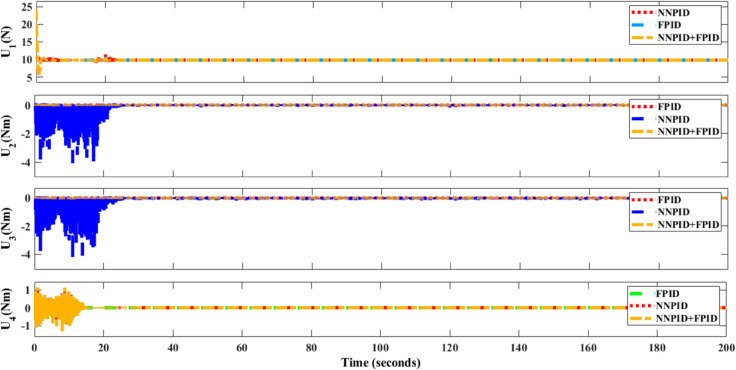
Control effort for helical trajectory tracking with input disturbance including hybrid controller.

**Fig 28:**
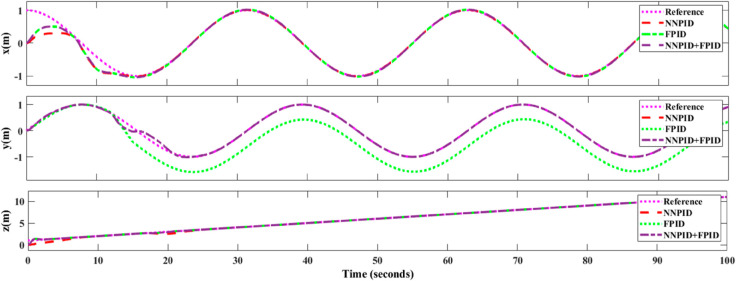
Position tracking response for helical trajectory tracking with input disturbance including hybrid controller.

**Fig 29:**
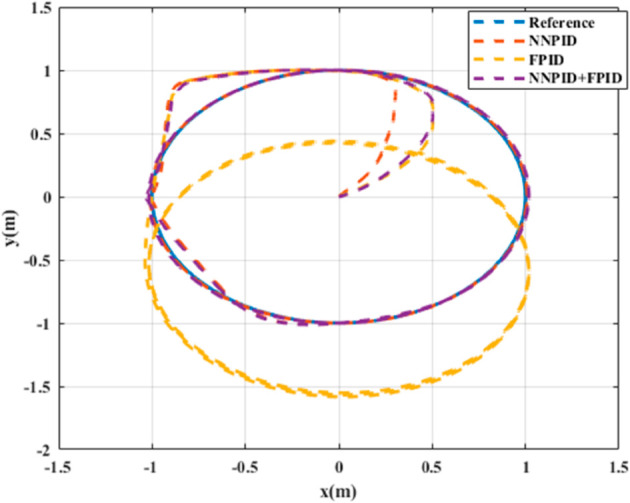
xy tracking response for helical trajectory tracking with input disturbance including hybrid controller.

**Fig 30:**
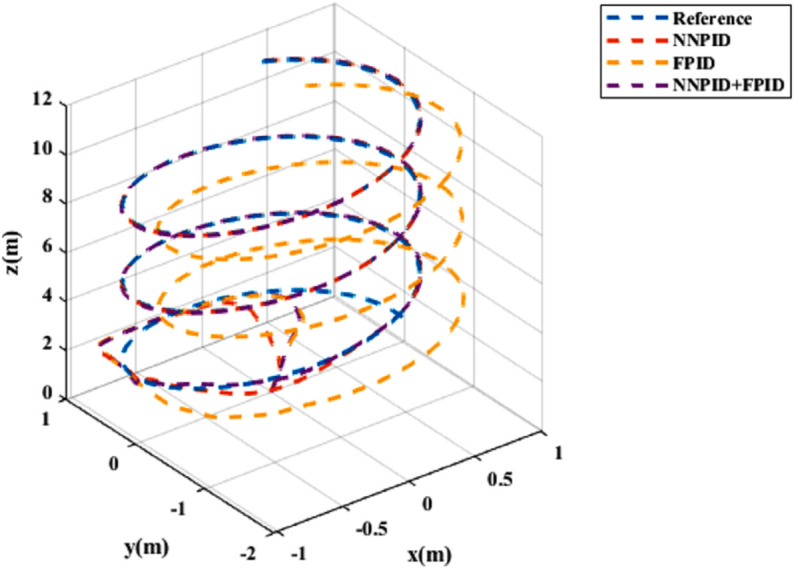
3D Plot for helical trajectory tracking with input disturbance including hybrid controller.

The simulation results in disturbed conditions for helical trajectory tracking including the hybrid approach are depicted in Figs 27, 28, 29, and 30.

### 8.2 Robustness check against variation in parameters

**Fig 31:**
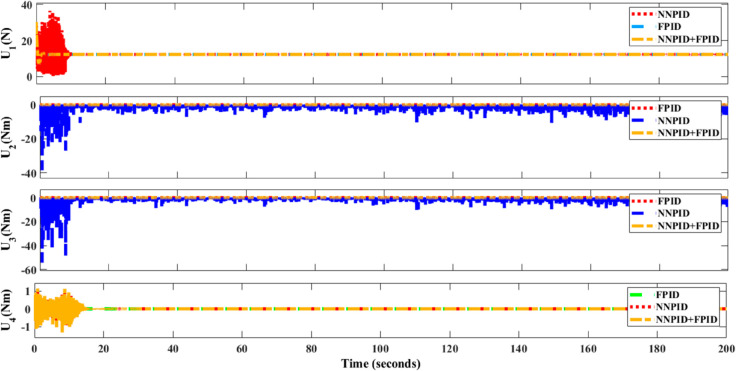
Control effort for helical trajectory tracking in the presence of parameter variation including hybrid controller.

**Fig 32:**
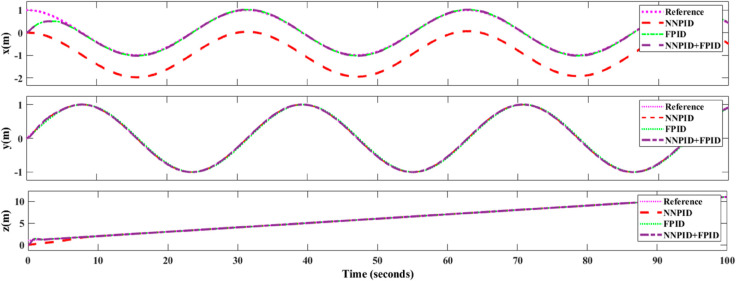
Position tracking response for helical trajectory tracking in the presence of parameter variation including hybrid controller.

**Fig 33:**
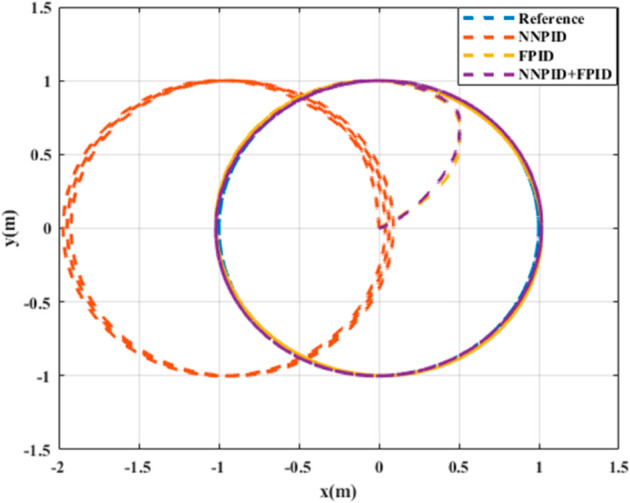
xy tracking response for helical trajectory tracking in the presence of parameter variation including hybrid controller.

**Fig 34:**
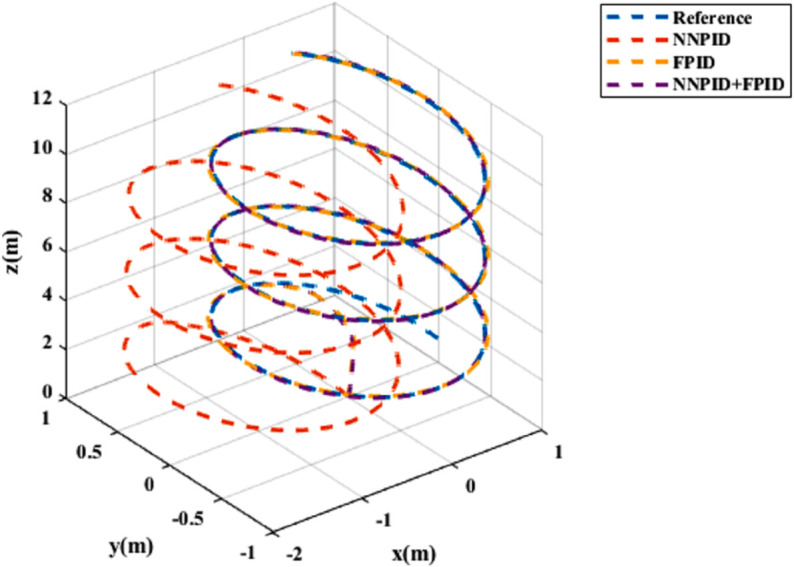
3D Plot for helical trajectory tracking in the presence of parameter variation including hybrid controller.

The simulation results for helical trajectory, incorporating the parameters variation are presented in Figs 31, 32, 33, and 34.

## 9 Comparison of controllers for helical trajectory based on performance criteria

Control system design plays a pivotal role in achieving precise trajectory tracking, especially when dealing with complex and continuous trajectories, like a helical trajectory. The efficiency of controllers in navigating disturbances and parameter variations while maintaining optimal performance is paramount. This study undertakes a comprehensive comparison of three controllers—Fuzzy Proportional-Integral-Derivative (FPID), Neural network Proportional-Integral-Derivative (NNPID), and a proposed controller, NNPID+FPID (hybrid control) approach based on performance criteria in the tabular form described in Tables 9, 10, and 11 in undisturbed conditions and disturbed (with input disturbance) and variation in parameters respectively.

**Table 9:** Comparison of Controllers Using MSE Criteria for helical Trajectory (undisturbed condition).

States	NNPID (MSE)	FPID (MSE)	NNPID+FPID (MSE)
*x*	5.561	3.041	1.051
*y*	$4.878\;\times \;10^{-5}$	0.003699	$4.878\;\times \;10^{-6}$
*z*	8.81	0.01709	0.00175
*ϕ*	$4.81\;\times \;10^{4}$	$8.96\;\times \;10^{-6}$	$6.87\;\times \;10^{-7}$
*θ*	1488	0.0007452	0.00006548
*ψ*	$2.315\;\times \;10^{-35}$	0.003558	$2.312\;\times \;10^{-35}$

**Table 10:** Comparison of controllers using MSE criteria for helical trajectory tracking in the presence of input disturbances.

States	NNPID (MSE)	FPID (MSE)	NNPID+FPID (MSE)
*x*	11.54	7.621	4.382
*y*	$4.38\;\times \;10^{-5}$	1208	$4.38\;\times \;10^{-6}$
*z*	18.63	0.1132	0.0114
*ϕ*	$4.95\;\times \;10^{4}$	$9.56\;\times \;10^{-6}$	$7.54\;\times \;10^{-7}$
*θ*	$1.0895\;\times \;10^{4}$	0.001306	0.00121
*ψ*	$2.35\;\times \;10^{-35}$	0.003558	$2.335\;\times \;10^{-35}$

**Table 11:** Comparison of controllers Using MSE criteria for helical trajectory tracking in the presence of parameter variation.

States	NNPID (MSE)	FPID (MSE)	NNPID+FPID (MSE)
*x*	4470	3.128	2.145
*y*	$4.38\;\times \;10^{-5}$	0.009486	$4.335\;\times \;10^{-5}$
*z*	12.21	0.01709	0.00185
*ϕ*	405.5	$2.06\;\times \;10^{-6}$	$2.16\;\times \;10^{-7}$
*θ*	348.5	0.000735	0.000467
*ψ*	$3.905\;\times \;10^{-35}$	0.003925	0.000425

### 9.1 Performance analysis and discussion

The performance of Neural Network PID (NNPID), Fuzzy Logic PID (FPID), and the hybrid NNPID+FPID controllers is analyzed based on the Mean Squared Error (MSE) across three scenarios:

1. Helical trajectory tracking under undisturbed conditions,
2. Helical trajectory tracking under input disturbances,
3. Helical trajectory tracking under parameter variations.

This section discusses the strengths and limitations of each controller, focusing on their ability to handle different UAV dynamics.

#### 9.1.1 Neural Network PID (NNPID)

#### Strengths

- **Exceptional precision in *y* and *ψ* states:**
  – In all scenarios, NNPID achieves remarkably low MSE for *y* and *ψ* states. For example, under undisturbed conditions, MSE for *y* ($4.878\;\times \;10^{-5}$) and *ψ* ($2.315\;\times \;10^{-35}$) indicates precise trajectory tracking.
  – This precision persists even under disturbances and parameter variations.
- **Robustness in non-linear dynamics:**
  – The adaptability of NNPID, driven by its neural network structure, makes it effective in handling the non-linear dynamics of UAV states such as *y* and *ψ*.

#### 9.1.2 Limitations

- **Poor performance in x,z,ϕ,θ states:**
  – NNPID struggles to achieve low MSE in x,z,ϕ,θ states, especially under parameter variations. For example, in the presence of parameter variations, *x* has an MSE of 4470, which is significantly worse than FPID and the hybrid approach.
- **Sensitivity to disturbances in certain states:**
  – States such as *x* and *z* experience performance degradation under disturbances. For example, in disturbed conditions, the MSE for *x* rises to 11.54 and for *z* to 18.63.

#### 9.1.3 Fuzzy Logic PID (FPID)

##### Strengths

- **Superior performance in x,z,ϕ,θ states:**
  – FPID consistently outperforms NNPID in these states. For example, under undisturbed conditions, FPID achieves lower MSE for *x* (3.041 vs. 5.561 for NNPID) and *z* (0.01709 vs. 8.81 for NNPID).
  – Similarly, for *ϕ* ($8.96\;\times \;10^{-6}$) and *θ* (0.0007452), FPID achieves results several orders of magnitude better than NNPID.
- **Resilience under disturbances and parameter variations:**
  – FPID demonstrates strong robustness to disturbances and parameter variations for x,z,ϕ,θ states. For instance, in disturbed conditions, FPID achieves an MSE of 7.621 for *x* compared to 11.54 for NNPID, and 0.1132 for *z* compared to 18.63 for NNPID.
- **Computational efficiency:**
  – FPID uses rule-based logic with pre-defined membership functions, resulting in simpler computations suitable for real-time control of states like x,z,ϕ,θ.

##### Limitations

- **Lower precision in *y* and *ψ* states:**
  – FPID has higher MSE for *y* and *ψ* states across all scenarios. For example, under undisturbed conditions, FPID’s MSE for *y* is 0.003699 compared to $4.878\;\times \;10^{-5}$ for NNPID.
- **Limited adaptability in highly non-linear states:**
  – FPID struggles to adapt to non-linear dynamics like *y* and *ψ* due to its reliance on heuristic rules, which lack the precision of neural networks.

#### 9.1.4 Hybrid Controller (NNPID+FPID)

##### Strengths

- **Best overall performance:**
  – The hybrid controller achieves the lowest MSE across all states in every scenario, combining the strengths of NNPID (precision for y,ψ) and FPID (robustness for x,z,ϕ,θ).
  – For example, under undisturbed conditions, the hybrid controller reduces *x* MSE to 1.051 (from 5.561 for NNPID and 3.041 for FPID) and *z* MSE to 0.00175 (from 8.81 for NNPID and 0.01709 for FPID).
- **Improved resilience to disturbances and parameter variations:**
  – The hybrid approach maintains robustness across all states under disturbances and parameter variations. For instance, in the presence of parameter variations, the hybrid controller achieves an MSE of 2.145 for *x*, compared to 4470 for NNPID and 3.128 for FPID.

##### Limitations

- **Higher computational cost:**
  – Combining NNPID and FPID increases computational requirements, which may be challenging for real-time implementation on resource-constrained systems.
- **Complex integration:**
  – The design and tuning of the hybrid controller require careful coordination between the two components to ensure seamless operation.

## 10 Conclusion and future work

This study evaluated the performance of Neural Network PID (NNPID), Fuzzy Logic PID (FPID), and their hybrid implementation (NNPID+FPID) for UAV helical trajectory tracking under undisturbed conditions, input disturbances, and parameter variations. The hybrid controller (NNPID+FPID) demonstrated superior performance by combining the precision of NNPID in *y* and *ψ* states with the robustness of FPID in *x*, *z*, *ϕ*, and *θ*. Despite higher computational demands, it provided superior tracking accuracy and resilience, making it the most effective solution for UAV control in complex dynamic environments.

Key findings include:

- **NNPID Controller:** Exhibited high precision in *y* and *ψ* states due to its adaptability to non-linear dynamics but showed weaker performance in *x*, *z*, *ϕ*, and *θ* states, particularly under parameter variations.
- **FPID Controller:** Demonstrated robustness and computational efficiency in *x*, *z*, *ϕ*, and *θ* states but lacked adaptability in handling the complex dynamics of *y* and *ψ* states.
- **Hybrid NNPID+FPID Controller:** Achieved the best overall performance, offering superior tracking accuracy, robustness, and adaptability across all states and scenarios, with the lowest mean squared error (MSE) compared to standalone controllers.
- **Computational Complexity:** The hybrid controller’s enhanced performance comes at the cost of increased computational demands and integration complexity.

In conclusion, the hybrid NNPID+FPID controller provides a robust and effective solution for UAV control, particularly in environments characterized by dynamic uncertainties, disturbances, and parameter variations.

### 10.1 Future work

Despite the promising results of the hybrid controller, several areas for future research can be explored to further enhance its performance and applicability: **Real-Time Implementation:** Testing the hybrid controller on physical UAV platforms in real-world conditions is essential to validate its effectiveness beyond simulations. **Advanced Learning Techniques:** Leveraging advanced neural network architectures, such as deep learning or reinforcement learning, can enhance adaptability and precision in dynamic or highly uncertain environments. **Adaptive Fuzzy Systems:** Incorporating self-tuning or adaptive fuzzy logic systems can enhance performance in non-linear dynamics, effectively bridging heuristic rules and learned behaviors. **Multi-Objective Optimization:** Developing a multi-objective optimization framework for the hybrid controller can achieve balanced performance metrics, such as trajectory accuracy, energy efficiency, and computational cost. **Extension to Multi-Agent UAV Systems:** Extending the hybrid control framework to coordinate multiple UAVs in dynamic environments presents opportunities for swarm robotics and collaborative mission applications.
